# Cross-Ring Fragmentation Patterns in the Tandem Mass Spectra of Underivatized Sialylated Oligosaccharides and Their Special Suitability for Spectrum Library Searching

**DOI:** 10.1007/s13361-018-2106-8

**Published:** 2018-12-18

**Authors:** Maria Lorna A. De Leoz, Yamil Simón-Manso, Robert J. Woods, Stephen E. Stein

**Affiliations:** 1 Mass Spectrometry Data Center, National Institute of Standards & Technology, 100 Bureau Drive Stop 8362, Gaithersburg, MD 20899 USA; 2 Present Address: Agilent Technologies, Inc., 2500 Regency Parkway, Cary, NC 27518 USA; 3 Complex Carbohydrate Research Center and Department of Biochemistry and Molecular Biology, University of Georgia, 315 Riverbend Road, Athens, GA 30602 USA

**Keywords:** Glycosylation, Sialylation, Cross-ring, Tandem MS, CID, Reference library, NIST glycan library

## Abstract

Reference spectral library searching, while widely used to identify compounds in other areas of mass spectrometry, is not commonly used in glycomics. Building on a study by Cotter and coworkers on analysis of sialylated oligosaccharides using atmospheric pressure-matrix-assisted laser-induced tandem mass spectrometry (MS/MS), we show that library search methods enable the automated differentiation of such sialylated oligosaccharide isomers using MS/MS derived from electrospray collision-induced dissociation in ion trap and beam-type fragmentation mass spectrometers. We compare MS/MS spectra of five sets of native sialylated oligosaccharide isomers and show a spectral library search method that can distinguish between these isomers using the precursor ion [M+2X-H]^+^, where X=Li, Na, or K. Sialic acid linkage (α2,3 vs. α2,6) is known to have a dramatic effect on the fragmentation of the sialylated compounds. We found that ^2,4^A3 cross-ring fragment at the terminal monosaccharide in sialyllactoses, sialyllactosamines, and sialyl pentasaccharides is highly abundant in the MS/MS spectra of [M+2X-H]^+^ species of α2,6-NeuAc glycans, while (^2,4^A3-H2O) fragment is highly abundant in α2,3-NeuAc moiety. The ^2,4^A3-H2O peak is specific to NeuAc-α2,3-Gal-β1,4-Y (Y=GlcNAc or Glc). To our knowledge, this observation was not reported previously. Theoretical calculations reveal major conformational differences between α2,6-NeuAc and α2,3-NeuAc structures that provide reasonable explanations for the observed fragmentation patterns. Other singly-charged ions ([M+X]^+^) do not show similar cross-ring cleavages. Implemented in a searchable library, these spectral differences provide a facile method to distinguish sialyl isomers without derivatization. We also found good spectral matching across instruments. MS/MS spectra and tools are available at http://chemdata.nist.gov/glycan/spectra.

**Electronic supplementary material:**

The online version of this article (10.1007/s13361-018-2106-8) contains supplementary material, which is available to authorized users.

**Figure:**
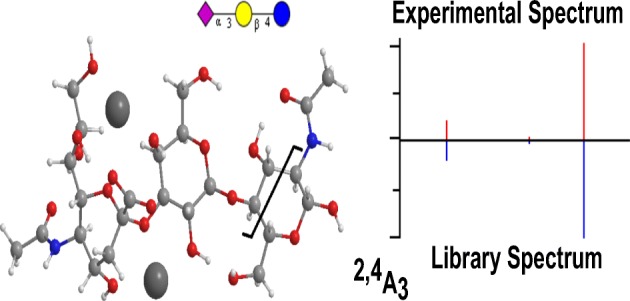
Graphical Abstract

## Introduction

Sialylated (or sialic acid-containing) oligosaccharides play a role in several biological functions, including host-pathogen interactions, cell protection from membrane proteolysis, cell adhesion, and cell-cell recognition [1, 2]. Sialic acids are nine-carbon acidic monosaccharides typically found in N-glycans, O-glycans, human milk oligosaccharides, and glycosphingolipids. *N*-Acetylneuraminic acid (abbreviated Neu5Ac, NeuAc or historically, NANA) is the most common sialic acid and is the only sialic acid naturally present in humans [3]. NeuAc is usually attached to terminal galactose (Gal) residues with an α2,3 or an α2,6 linkage or to terminal *N*-acetylglucosamine (GlcNAc) residues with an α2,6 linkage. In human milk oligosaccharides, NeuAc can also be found attached to the internal Gal or GlcNAc residues [4].

Distinguishing sialyl linkage isomers is critical because most biological activities of sialyl oligosaccharides are structure-dependent [5]. For example, α2,3-sialyllactose, a human milk oligosaccharide, binds to *Helicobacter pylori*, effectively lessening the adhesion of the bacteria to duodenum-derived human cells [6]. The human influenza virus recognizes α2,6-sialyl linkages; the avian and equine influenza virus recognizes the α2,3-sialyl linkages; and the swine influenza virus seems to recognize both α2,3 and α2,6 sialyl linkages [7]. In biomarker discovery, increased α2,6-sialylation was found to be correlated in the progression of many types of cancer [8].

Using matrix-assisted laser desorption/ionization-time of flight (MALDI-TOF) MS in the negative ion mode, Yamagaki and Nakanishi [5] distinguished α2,3 and α2,6 sialyl isomers of sialyllactose (SL) and sialyllactosamine (SLN) using difference in relative intensities of the B_1_ fragment ion (*m/z* 290) (for a detailed explanation of the fragmentation nomenclature, see reference [9]). The α2,3-sialic acid cleaves easier in MALDI-post-source decay (PSD) fragmentation, resulting in higher intensities of the B_1_ ion. Tandem mass spectrometry using soft ionization techniques, such as MALDI and electrospray (ESI), is a powerful tool to analyze glycans. However, sialic acid is labile and may be lost by in-source or metastable decay in MALDI-MS [10]. In positive-ion ESI-MS, the tandem mass spectra of the protonated or sodiated species of underivatized sialylated glycans usually contain very few peaks since the main fragmentation comes from the loss of the sialic acid [11].

The structural characterization of glycans released from proteins follows different approaches, usually implicating labeling, derivatization, and others. Derivatizations, such as permethylation [12, 13], amidation [14], and esterification [15–18] and are useful in stabilizing sialic acids for analysis in the positive ion mode and when combined with multi-stage MS (MS^n^), may give valuable cross-ring cleavage peaks and open hydroxyl scars to help elucidate the full glycan structure [19]. However, derivatization may be incomplete and time-consuming. Underivatized sialylated glycans analyzed in the negative ion mode ESI-MS generally produce better fragmentation [20], but the MS analyses are typically harder to optimize. Cotter and coworkers reported that by using infrared-atmospheric pressure (IR-AP) MALDI-ion trap MS with glycerol as matrix, they were able to distinguish cationized sialylated isomers in their underivatized form [11]. They found that doubly sodiated or cobaltinated singly-charged underivatized sialyl glycans produced distinct spectra with cross-ring cleavages.

Building on Cotter’s work, we report spectra with cross-ring cleavages of underivatized sialyl glycans acquired by collision-induced dissociation (CID) in ion trap (IT) and beam-type fragmentation (CID MS/MS, higher-energy collision dissociation (HCD) MS/MS, and CID MS^n^) at several collision energies using ESI-Orbitrap and ESI-quadrupole TOF (QTOF) MS instruments. We surveyed several precursor ions and confirmed that the [M + 2X-H]^+^ (where X = Li, Na, or K) precursor ions produce cross-ring-rich tandem mass spectra, as Cotter reported earlier using AP MALDI MS [11]. We show that using ESI ionization, these fragment ions help differentiate sialyl isomers without the need for derivatization or online purification. The signals are intense with minimal adverse effects of the metal ion salts on the ion signals. We find good spectral matching in the IT CID, QTOF, and HCD Orbitrap MS/MS data across several collision energies. Thus, the analysis of [M + 2X-H]^+^ ions with library searching could be used to differentiate sialyl isomers without derivatization. However, such ions are generally lesser in abundance compared to [M + Na]^+^ ions, so further work on optimizing the ionization efficiency of these ions is necessary.

In addition to the identification of specific glycans, a goal of this work to show the effectiveness of a library searching for aiding the identification of glycans, especially by distinguishing isomers having different fragmentation patterns.

## Materials and Methods

### Materials

Twelve oligosaccharides, such as 3′-sialyllactose (3-SL), 6′-sialyllactose (6-SL), 3′-sialyl-*N*-acetyllactosamine (3-SLN), 6′-sialyl-*N*-acetyllactosamine (6-SLN), sialyllacto-*N*-tetraose a (LSTa), sialyllacto-*N*-tetraose b (LSTb), sialyllacto-*N*-tetraose c (LSTc), sialyllacto-*N*-tetraose d (LSTd), 3′-sialyl-Lewis A tetrasaccharide (SLeA), 3′-sialyl-Lewis X tetrasaccharide (SLeX), sialylated tetraose type 1 (STetra1), and sialylated tetraose type 2 (STetra2), were obtained from Sigma-Aldrich (St. Louis, MO), V-labs (Covington, LA), and Elicityl (Crolles, France). All materials were used without additional purification.

### Mass Spectrometry of Oligosaccharides

The underivatized glycans were prepared with, and without, dopants. Cation-doped diluted solution (75:25 water/methanol) were prepared by adding lithium iodide, sodium chloride, or potassium iodide to obtain lithiated, sodiated, or potassiated precursor ions, respectively, prior to MS analysis. Samples were analyzed by direct infusion using a nano-ESI LTQ-Orbitrap Velos or LTQ-Orbitrap Elite MS (Thermo Fisher Scientific, Waltham, MA). Samples were infused at a flow rate of 50 to 100 nL/min using equal volumes of aqueous methanol or aqueous acetonitrile. Data were acquired in the positive ionization mode at a mass range of *m/z* 100 to *m/z* 1500. Full-scan Fourier-transform (FT) mass spectra were acquired at a resolution of 30,000. Default values were used for the activation Q and time. CID MS/MS and MS^n^ spectra were obtained at 35% normalized collision energy and with an isolation width of 2 Da. FT CID and HCD MS/MS spectra were obtained at a resolution of 15,000 and 30,000, respectively. HCD MS/MS spectra were acquired at several normalized percent collisional energies ranging from 5 to 180%, but only the useful spectra were included in the library. For example, spectra with a single ion peak are usually not included in the library. Calibration was done using a calibrant mix provided by the manufacturer to allow mass accuracy of 10 ppm (ppm)^1^ or better over the entire *m/z* range. Oligosaccharides were detected as [M + X]^+^, [M + 2X-H]^+^, [M + 2X]^2+^, [M + X + H]^2+^, and [M + H]^+^ precursor ions, where M = molecular ion and X = Li, Na, or K, depending on the dopant.

Similar spectra of a particular glycan acquired using the same instrument, activation method, and collision energy were clustered together using a stringent clustering algorithm in order to generate a consensus spectrum [21, 22]. Several consensus spectra were generated for each glycan. Multi-stage MS (MS^n^) was performed when further characterization was necessary.

### Theoretical Calculations

Three-dimensional (3D) structures were built using GLYCAM-web (www.glycam.org). The structures were preoptimized using molecular mechanics as implemented in Amber16 [23] using the GLYCAM force field [24, 25] with the most recent version of the GLYCAM06 parameters [25]. Although no systematic exploration of the conformational space was performed, the potential surface was spanned starting from various initial configurations. Subsequently, quantum mechanical calculations were performed using the density functional theory (DFT) method with the B3LYP functional and either standard 6-31G(d) or LANLDZ basis sets as implemented in Gaussian 09 [26]. Several relaxed scans were performed for exploring potential fragmentation pathways. Similar calculations have been effectively used in previous studies of ion fragmentation under CID conditions [27, 28]. Frequency analyses at the same level of theory were performed to identify real minima and transition state structures on the potential energy surface, for the model structures, 3-SLN and 6-SLN. Geometry optimization of more complex structures, such as LSTa and LSTc, were performed using the semi-empirical Hamiltonian AM1.

## Results and Discussion

### 3-Sialyllactose (3-SL) Versus 6-SL

**Figure 1:**
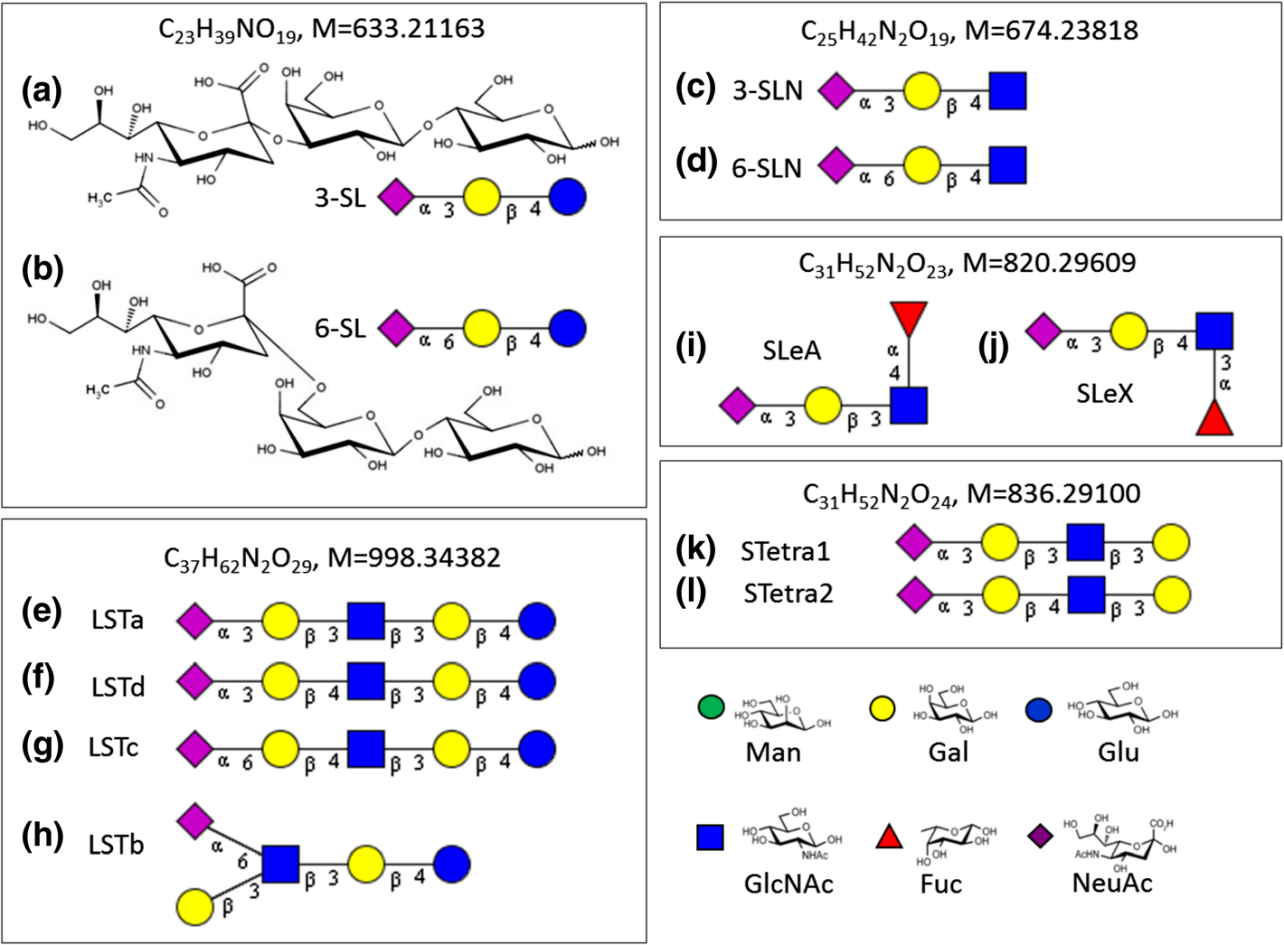
Structures of analyzed sialyloligosaccharides. (**a**) 3′-Sialyllactose (3-SL), (**b**) 6′-sialyllactose (6-SL); cartoon representations of (**c**) 3′-sialyl-*N*-acetyllactosamine (3-SLN), (**d**) 6′-sialyl-*N*-acetyllactosamine (6-SLN); (**e**) sialyllacto-N-tetraose a (LSTa), (**f**) sialyllacto-*N*-tetraose d (LSTd), (**g**) sialyllacto-*N*-tetraose c (LSTc), (**h**) sialyllacto-*N*-tetraose b (LSTb), (**i**) 3′-sialyl-Lewis A tetrasaccharide (SLeA), (**j**) 3′-sialyl-Lewis × tetrasaccharide (SLeX), (**k**) sialylated tetraose type 1 (STetra1), and (**l**) sialylated tetraose type 2 (STetra2). Each box is a set of isomers. Symbol nomenclature based on the recommendations by the consortium for functional glycomics (yellow circle: galactose (Gal), blue circle: glucose (Glc), blue square: *N*-acetylglucosamine (GlcNAc), red triangle: fucose (Fuc), purple diamond: *N*-acetylneuraminic acid (NeuAc)

We compared the consensus [21, 22] tandem mass spectra of SL isomers shown in Figure 1A and B. The two isomers differ in the sialic acid linkage, with Figure 1A having an α2,3-linked NeuAc (3-SL) and Figure 1B having an α2,6-linked NeuAc (6-SL).

**Figure 2:**
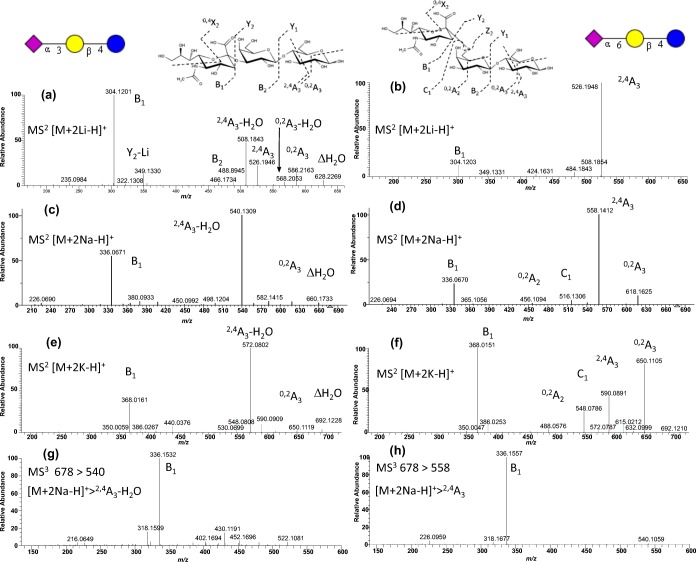
FT CID MS/MS spectra of [M + 2X-H]^+^ ion of 3-SL (left) and 6-SL (right), where X is (**a**) lithium adducted to 3-SL (*m/z* 646.24); (**b**) lithium adducted to 6-SL (*m/z* 646.24); (**c**) sodium adducted to 3-SL (*m/z* 678.18); (**d**) sodium adducted to 6-SL (*m/z* 678.18); (**e**) potassium adducted to 3-SL (*m/z* 710.13); or (**f**) potassium adducted to 6-SL (*m/z* 710.13). Positive FT CID MS^3^ spectra of (**g**) ^2,4^A_3_-H_2_O from [M + 2Na-H]^+^ ion of 3-SL (*m/z* 540.13) and **h**^2,4^A_3_ from [M + 2Na-H]^+^ ion of 6-SL (*m/z* 558.32)

In the native form, small oligosaccharides are typically analyzed using the precursor ion [M + Na]^+^. The CID MS/MS of the [M + X]^+^ ion (where X = Li, Na, or K) of 3-SL and 6-SL trisaccharides showed primarily the Y_2_ ion (loss of the labile sialic acid) in all cases and provided minimal differences in the spectra of the isomers (see Supplemental Figure 1). However, the spectra of [M + 2X-H]^+^ precursor ions of 3-SL and 6-SL isomers gave distinct tandem mass spectra enough to differentiate the isomeric pairs, a difference observed previously by Cotter and coworkers using AP-MALDI ion trap MS [11]. Figure 2A–F shows the MS/MS FT CID spectra of [M + 2X-H]^+^ ion (where X = Li, Na, K) of 3-SL (left) and 6-SL (right). In all cases, the greatest difference was the intensities of the cross-ring cleavage pair of the reducing glucose, namely ^2,4^A_3_ and (^2,4^A_3_-H_2_O). The α2,6-linked SL favors the formation of the ^2,4^A_3_ product ion, while the α2,3-linked SL favors the formation of ^2,4^A_3_-H_2_O product ion. Upon closer inspection, we see that the different cation-bound ions show different behaviors. The α2,-6-linked [M + 2Li-H]^+^ ion favors the formation of the ^2,4^A_3_ ion at 100% intensity; the B_1_ ion is at < 20% intensity (Figure 2B). The α2,3-linked [M + 2Li-H]^+^ ion favors the formation of the B_1_ ion (100%); the ^2,4^A_3_-H_2_O ion is at ≈ 50%; the ^2,4^A_3_ ion is at > 20%; and the ^0,2^A_3_ ion is at ≈ 10% (Figure 2A). The corresponding sodiated ion [M + 2Na-H]^+^ of the α2,6- and α2,3-linked SL favors the ^2,4^A_3_ and ^2,4^A_3_-H_2_O, respectively, at 100% intensity (Figure 2C and D). The [M + 2Li-H]^+^ ion of the α2,3-linked species favors the ^2,4^A_3_-H_2_O ion formation at 100% (Figure 2E), while the α2,6-linked species only favors the ^2,4^A_3_ formation at < 50% (Figure 2F).

MS^3^ analysis of the ^2,4^A_3_ ion (*m/z* 540) from [M + 2Na-H]^+^ of 3-SL reveals primarily the B_1_ ion (sialic acid), *m/z* 430, and *m/z* 522, as shown in Figure 2G. MS^3^ analysis of ^2,4^A_3_-H_2_O (*m/z* 558) from [M + 2Na-H]^+^ of 6-SL (Figure 2H) also gave the B_1_ ion (sialic acid) as the base peak, but the other small peaks from Figure 2G were not observed, including *m/z* 430.

The ^2,4^A_3_ ion is a cross-ring cleavage in the reducing glucose. The two isomers only differ in the linkage of the sialic acid, a residue that is two monosaccharides away from the cross-ring cleavage site. The fact that the cross-ring cleavages are not observed in the singly sodiated precursor ion means that the two sodium ions and the loss of H^+^ help in the overall stability of the glycosidic bonds. Without the labile glycosidic bonds, the cross-ring cleavages become more abundant [11]. Most likely, one sodium ion replaces the proton in the carboxylic acid, accounting for one sodium gain and one proton loss. The assignment of the other sodium could be challenging [11]. Most fragments are observed as sodium adducts.

### 3-Sialyllactosamine (3-SLN) Versus 6-SLN

**Figure 3:**
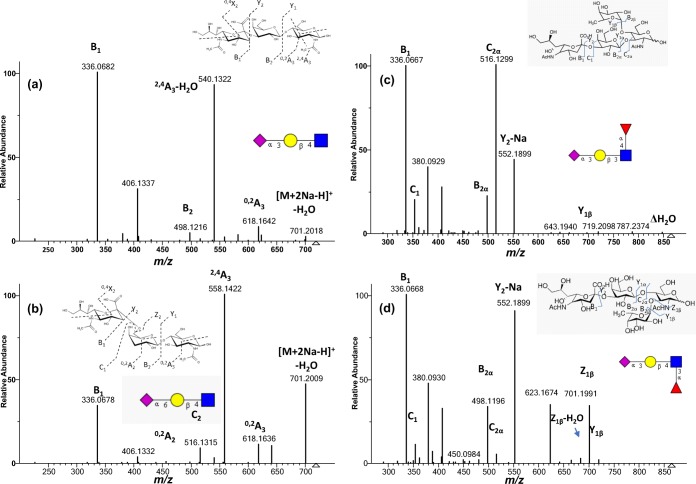
Consensus FT CID MS/MS spectra of [M + 2Na-H] + ion of SLN isomers (*m/z* 719.2097): (**a**) 3-SLN and (**b**) 6-SLN; and sialyl Lewis isomers (*m/z* 865.2672): (**c**) SLeA and (**d**) SLeX. Consensus spectra from the NIST 17 tandem MS library (http://chemdata.nist.gov)

To verify if the differences in spectra of [M + 2Na-H]^+^ precursor ions of 3-SL and 6-SL were also applicable to other oligosaccharides, we probed another sialyl trisaccharide pair, 3-SLN (Figure 1C) and 6-SLN (Figure 1D), differing from 3-SL and 6-SL only in the reducing monosaccharide residue, GlcNAc. Figure 3 shows the [M + 2Na-H]^+^ spectra of 3-SLN (Figure 3A) and 6-SLN (Figure 3B). Table 1 summarizes the fragmentation observations for [M + 2Li-H]^+^, [M + 2Na-H]^+^, and [M + 2 K-H]^+^ ions. Following the same pattern in the SL isomers, the cross-ring cleavage pair in the reducing GlcNAc was observed: the ^2,4^A_3_-H_2_O peak at *m/z* 540 is ≈ 100% intensity in 3-SLN but is only < 5% intense in 6-SLN and the ^2,4^A_3_ peak at *m/z* 558 is the base peak in 6-SLN but is only < 5% intense in 3-SLN. Also, the B_1_ ion is the base peak in 3-SL but only ≈ 40% intense in 6-SL.

**Table 1:** Relative Fragment Ion Abundances^*^ in the MS/MS Spectra of α2,3- and α2,6-sialyllactosamines and the Four LST Isomers

Isomer	Ion	B1	^0,2^A_3_	^2,4^A_3_-H_2_O	^2,4^A_3_	^2,4^A_5_-H_2_O	^2,4^A_5_
3 (6)-SLN	[M + 2Li-H]^+^	100 (10)	25 (100)	50 (< 1)	15 (< 1)		
3 (6)-SLN	[M + 2Na-H]^+^	52 (25)	< 1 (100)	100 (< 1)	< 1 (15)		
3 (6)-SLN	[M + 2 K-H]^+^	33 (100)	< 1 (45)	100 (< 1)	2 (85)		
LSTa	[M + 2Na-H]^+^			< 1	< 1	15	10
LSTb	[M + 2Na-H]^+^			< 1	< 1	n/a	n/a
LSTc	[M + 2Na-H]^+^			< 1	< 1	< 1	100
LSTd	[M + 2Na-H]^+^			25	< 1	25	< 1

Legend: 3 (6)-SLN: 3′-sialyllactosamine (6′-sialyllactosamine), LST: sialylpentasaccharides *relative to the intensity of the most intense peak

**Figure 4:**
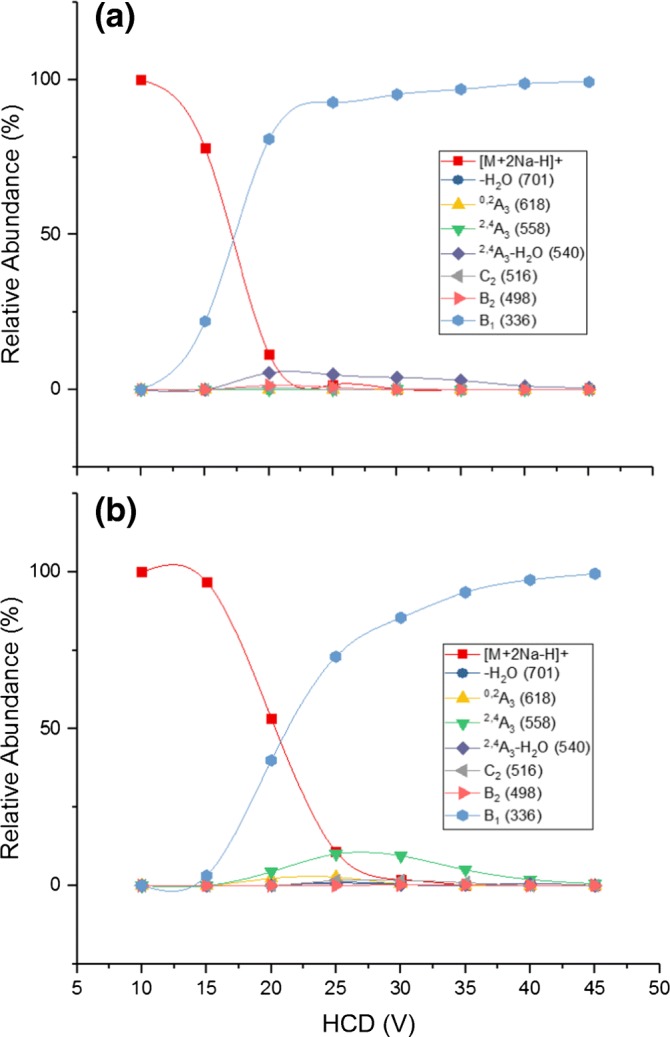
Product ion peak intensities as a function of the HCD normalized collision energy in the MS/MS spectrum of (**a**) 3-SLN and (**b**) 6-SLN. Each curve is identified by the *m/z* value of the corresponding production

Figure 4 shows product ion peak intensities as a function of the HCD normalized collision voltage in the MS/MS spectrum of 3-SLN (Figure 4A) and 6-SLN (Figure 4B) in the Orbitrap MS. For both isomers, the two major parallel fragmentation channels are the loss of sialic acid (full blue circles) and the cross-ring cleavages, ^2,4^A_3_ (full green triangles), but the latter is accompanied by a simultaneous loss of water (full purple diamonds). The loss of water in the reducing end from the precursor ion [M + 2Na-H]^+^ at *m/z* 701 is more intense in 6-SL than in 3-SL, whereas *m/z* 406 (Y_2_) is more intense in 3-SL.

Figure 4A and B demonstrates that around 20 V, the precursor ion intensities for both isomers have been reduced by approximately 50%. At collision energies below this voltage, only the B_1_ fragmentation is important. Also, the abundance of product ions from the cross-ring cleavages is only significant at normalized collision energies above this voltage. The abundance of cross-ring fragment ions, ^2,4^A_3_-H_2_O, for 3-SLN and ^2,4^A_3_ for 6-SLN, can reach up to 15% of the total ion abundance.

**Figure 5:**
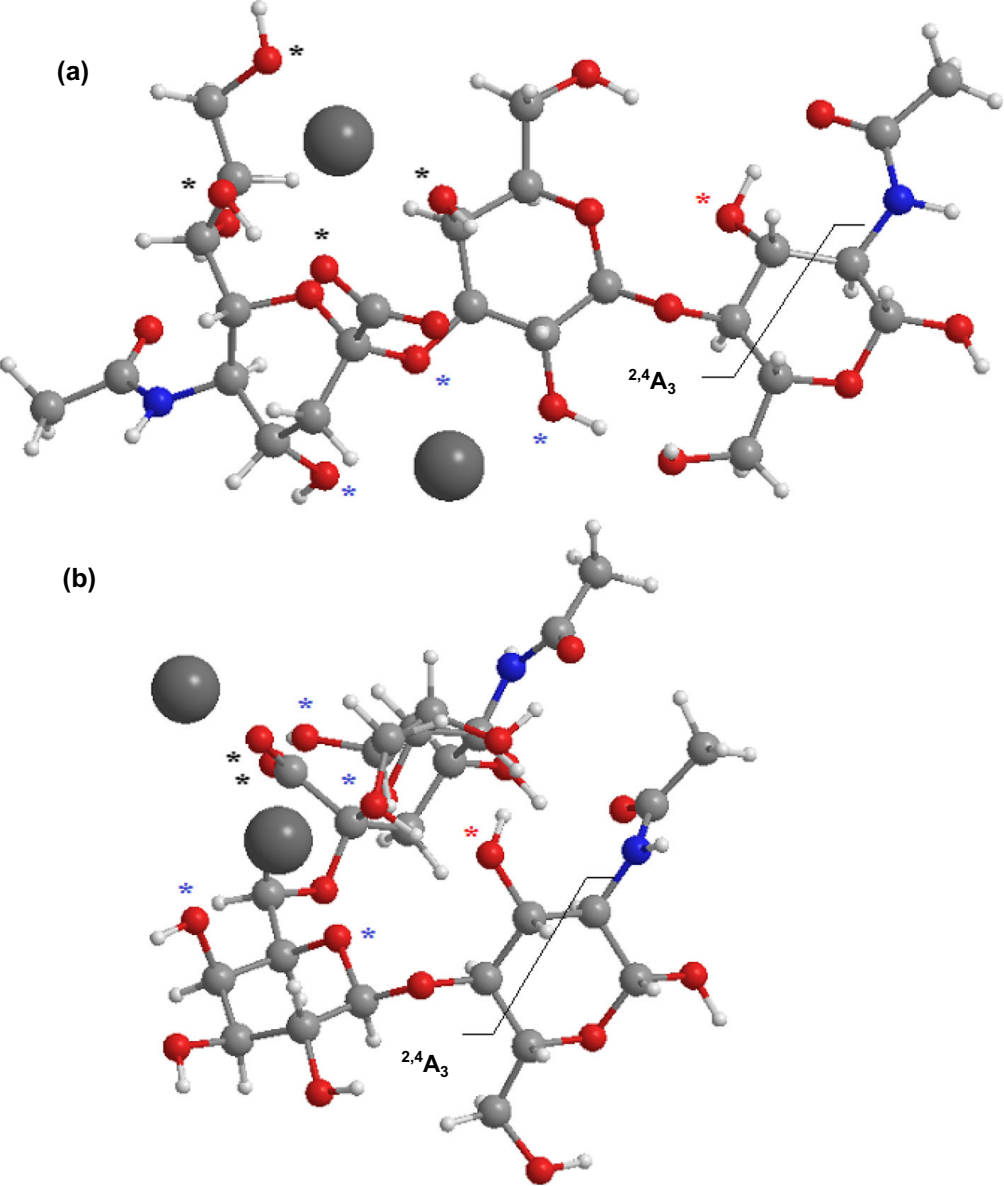
DFT optimized structures of 3-SLN (**a**) and 6-SLN (**b**), calculated at the B3LYP-LandlDZ level of theory, and showing the ^2,4^A_3_ cross-ring fragmentation. Black (blue) asterisks mark the oxygen atoms that are coordinated to the sodium ion in front (behind) the plane of the sugar at the reducing end. Red asterisks mark the oxygen atoms involved in the water losses from the ^2,4^A_3_ fragment ions

**Scheme 1:**
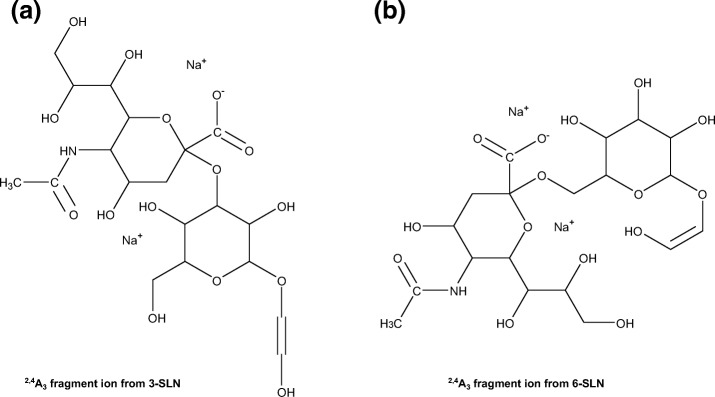
Planar representation of the ^2,4^A_3_ fragment ions from (**a**) 3-SLN and (**b**) 6-SLN. Figure 2 of the Supplementary information shows the tridimensional structures of the fragment ions

**Figure 6:**
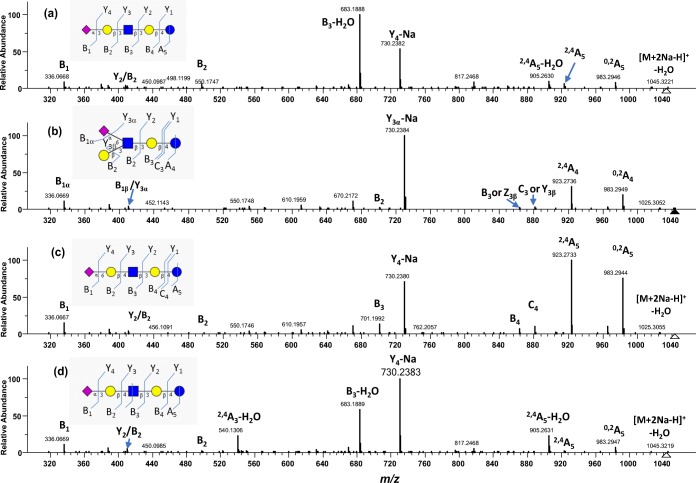
Consensus FT CID MS^2^ spectra of [M + 2Na-H]^+^ of four LST isomers (*m/z* 1043.31): (**a**) LSTa (*N* = 17), (**b**) LSTb (*N* = 33), (**c**) LSTc (*N* = 31), and (**d**) LSTd (*N* = 49). Consensus spectra from the NIST 17 tandem MS library (http://chemdata.nist.gov)

An exploratory theoretical study of the gas phase potential energy surface (PES) was conducted to find the most stable locations for the sodium ions using Amber 16. Several local minima were identified and re-optimized using the semi-empirical Hamiltonian AM1. Finally, geometry optimization of the “global” minima for both isomers was performed using DFT calculations. It showed a crucial structural disparity in the conformational structure of both isomers that explains, in part, the differences observed in the fragmentation patterns. Figure 5 shows the optimized structures of the [M + 2Na-H]^+^ ions of 3-SLN (Figure 5a) and 6-SLN (Figure 5b). It seems that in the gas phase, the positions of the sodium ions are determined by the carboxyl group of the sialic acid. One of the sodium ions is forced in the vicinity of the carboxyl group and coordinating other hydroxyl groups and the other sodium ion is located in the opposite side also coordinating several oxygens. In the 3-SLN isomer, for example, a sodium ion is about the same distance of 2.5 Å from one of the carboxyl oxygen and other three hydroxyl oxygens marked with black asterisks in Figure 5a. The second sodium ion is on the opposite side between the sialic acid and the second-sugar ring making the structure very rigid (the oxygen involved are marked with blue asterisks). A frequency calculation shows weak out-of-plane vibrational modes. On the other hand, in the 6-SLN, both sodium ions are also near the sialic acid end, but the α2,6-sialyl linkage allows the molecule to bend on itself hydrogen-bonding the sugar of the reducing end with the sialic acid. These structures explain the higher stability of the glycosidic bonds and the labile character of the cross-ring fragment. The computational modeling shown in Supplemental Figure 2 shows two major differences in the fragmentation patterns of the 3- and 6-SLN isomers. First, the loss of water from the precursor ions occurs from the C6 branch of the reducing end in both isomers; however, relaxed scans of the C-O bonds corresponding to these water losses show different behaviors for each isomer, consistent with the experimental observations. Whilst the loss of water occurs with a relatively low barrier from 6-SLN (≈ 80 kJ/mol), the stretching of the C▬O bond for the 3-SLN isomer requires more energy (≈ 120 kJ/mol) and at the same time promotes the ^2,4^A_3_ cleavage. (It is worth mentioning that our assumption that the loss of water is originated from the C6-branch is based calculations, looking for the minimum energy pathway in all possible water losses; however, it is also consistent with the spectra of both isomers; the B_1_ peak is less intense for the 6-SLN isomer that experiences the larger water loss.) Second, the ^2,4^A_3_ fragmentation of the extended structure of 3-SLN produces an ion that simultaneously experiences loss of water from the generated terminal residue (^2,4^A_3_ fragment ion structures are shown in Supplemental Figure 2; red asterisks in Figure 5 mark the oxygen atoms involved in the water losses from the ^2,4^A_3_ ions; an approximate planar representation of the structures of the fragment ions is shown in Scheme 1).

On the other hand, the 6-SLN’s bent structure favors to retain the water from the generated terminal residue. Of course, this is an oversimplified view of the fragmentation process, but an attempt to include the various contributions of different local minima assuming a Boltzmann distribution complicates matters and sheds no new light on the problem. The optimized coordinates of the molecular geometries are given in Supplemental Table 1. It seems also that the B_1_ peak is less abundant for the 6-SLN isomer due in part to loss of water from the sialyl moiety.

### Sialyl Lewis A (SLeA) Versus SLeX Spectra

Two other isomer pairs, SLeA (Figure 1I) and SLeX (Figure 1J), were examined. Both tetrasaccharide isomers contained an α2,3-linked sialic acid and differed in the lactosamine and fucosyl linkages. In Figure 3C and D, their spectra showed both glycosidic and cross-ring cleavages. The main differences are the intensities of Y_2_-Na (more abundant in SLeX) and C_2α_ (more abundant in SLeA) peaks and the presence of *m/z* 623 only in the SLeX spectrum.

The fact that C_2α_ is the base peak in SLeA suggests that the Gal-β1,3-GlcNAc bond is easier to break than the labile fucose or sialic acid. For SLeX, the Gal-β1,4-GlcNAc bond is harder to break. Instead, the labile sialic acid is easier to break, as observed in the base peak intensity of Y_2_-Na. This also shows that one of the sodium atoms sits on the sialic acid.

The fragmentation spectra of glycans with fucosyl linkages is dominated by glycosidic cleavages and the α2,3 effect is barely observed. The peak at *m/z* 623 in the spectrum of SLeX corresponds to loss of 242 Da, and it was initially attributed to the neutral ion pair of sodium ion and the product ion generated from cross-ring cleavage of sialic acid (^1,5^X_2_). However, the MS^3^ spectrum of the ion at *m/z* 623 shows a prominent B_1_ fragment ion (https://chemdata.nist.gov/glycan/). A previous report [29] showed that protonated native sialyl Lewis tetrasaccharide alkyl glycosides undergo internal-residue rearrangements with the fucose residue migrating toward the non-reducing terminal sialic acid residue. These authors reported two major unexpected peaks in the spectra of SLeA and SLeX at *m/z* 438 and *m/z* 600 of the [M + H] ^+^ ions, and they also mentioned that the abundance of these ions is significantly reduced for other ion types. In the present study, we observe an unexpected peak at *m/z* 623 in the spectrum of SLeX, but not in the spectrum of SLeA. Also, the presence of an intact B_1_ ion in the MS^3^ spectrum provides strong evidence against the fucosyl rearrangement mechanism. This is an important issue, because rearrangements can isomerize the sequences and lead to errors in interpreting oligosaccharide structures.

Instead of a cross-ring fragmentation of sialic acid or a rearrangement, there is another plausible explanation for the observed peaks in the spectrum of SLeX. The spectrum shows a prominent peak at *m/z* 701 corresponding to a loss of 164.0681 Da (the exact mass of fucose, 164.0685 Da). It seems that the peak at *m/z* 623 is originated from this peak by neutral loss of 78 Da {C_2_H_4_O_2_+ H_2_0}. According to Lebrilla et al. [30], this loss is characteristic in the spectra of negative ions of 1–4-linked disaccharides and involves the loss of the anomeric carbon atom and the adjacent carbon atom. This was first suggested by Garozzo et al. [31]. In the present case, the exact mass analysis and theoretical calculations suggests the loss of C_2_H_4_O_2_+ H_2_0 from the ion at *m/z* 701 (a planar representation of the fragmentation process is included with the supplementary information, Scheme S2). An experimental validation of these findings or an explanation for the observe differences with the SLeA isomer would require further investigation.

### MS/MS Spectral Comparison of LSTa, LSTb, LSTc, and LSTd Sialyl Pentasaccharides

Next, we analyzed four isomers of sialylated pentasaccharides (Figure 1E–H). Figure 6 shows the MS^2^ consensus spectra of the [M + 2Na-H]^+^ precursor ions at *m/z* 1043. LSTc (Figure 6C and LSTb (Figure 6B) are both α2,6-linked sialyl oligosaccharide, differing only in the point of attachment of the sialyl residue: the NeuAc residues of LSTc and LSTb are linked to Gal and GlcNAc, respectively. Visually, the spectral peaks of the two isomers differ only in intensities. This is a good example of where in-silico libraries would NOT work because peak intensities could help differentiate the two isomers. Empirical or experimental spectral libraries, such as the NIST MS/MS library take into account all fragmentation pattern differences, not only B_1_, cross-ring, or theoretical fragmentations as mentioned in previous work [5]. Even the cumulative effect of relatively small peak intensity, differences can render significantly different scores.

Moreover, as expected from the cross-ring cleavage patterns of the trisaccharides studied above, the cross-ring fragment of interest is an A cross-ring at the reducing end. For LSTc, this is the ^2,4^A_5_ cross-ring ion (Figure 6C) and for LSTb, it is the ^2,4^A_4_ ion (Figure 6B), both at *m/z* 923.

In LSTc shown in Figure 6C, the most intense peak is the ^2,4^A_5_ cross-ring ion, followed by another cross-ring fragment ^0,2^A_5_ and the loss of sialic acid (Y_4_-Na). In LSTb (Figure 6B), the most intense peak is the loss of sialic acid followed by the ^2,4^A_4_ cross-ring cleavage at the reducing glucose.

**Figure 7:**
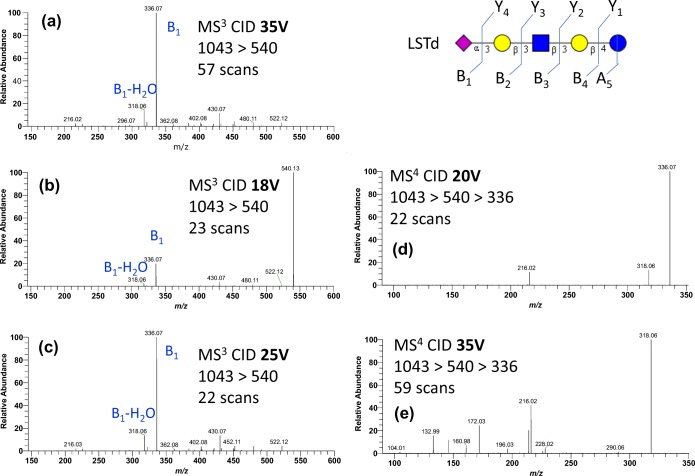
MS^n^ of product ions of [M + 2Na-H]^+^ of LSTd: (**a**) 35-V CID MS^3^ spectrum (*N* = 57) of *m/z* 540, (**b**) 18-V CID MS^3^ spectrum (*N* = 23) of *m/z* 540, (**c**) 25-V CID MS^3^ spectrum (*N* = 25) of *m/z* 540, (**d**) 20-V CID MS^4^ spectrum (*N* = 36) of *m/z* 336, and (**e**) 35-V CID MS^4^ spectrum (*N* = 59) of *m/z* 336

Figure 6C and D shows the [M + 2Na-H]^+^ CID MS^2^ spectra of LSTa and LSTd, respectively. Both isomers have an α2,3-linked sialic acid and they differ only in one lactosamine linkage: β1,3 for LSTa and β1,4 for LSTd. The peaks mostly differ in intensities except for one peak *m/z* 540 that only occurs at ≈ 30% intensity in the LSTd spectrum. Further examination of this peak by MS^3^ at 35 V FT CID (Figure 7A) shows that the spectrum matches the IT CID spectrum of 3-SL *m/z* 540 in Figure 2G.

The fact that the *m/z* 540 peak is observed in LSTd and not in LSTa means that this peak is not only specific to an α2,3-sialyl linkage [11], but it is specific to an α2,3-β1,4 linkage. To our knowledge, this observation was not reported previously.

Moreover, the FT CID spectrum of LSTa (Figure 6A) shows the preference of the formation of B_3_-H_2_O ion, while the LSTd spectrum (Figure 6D) shows preference of the Y_4_-Na ion. Following the pattern of SLs and SLNs, the ^2,4^A_5_-H_2_O ion is more abundant than the ^2,4^A_5_ ion. However, the difference in intensities is less than for smaller trisaccharides.

The CID MS/MS was activated at lower energies for the same precursor ion in order to understand the product ions. In Figure 7B, MS^3^ of the precursor ion *m/z* 540 at 18 V of CID energy shows that the said ion is the base peak, and the B_1_ ion is the second most abundant peak. We also see that *m/z* 318 and *m/z* 430 are starting to form. At 25 V of CID energy in Figure 7C, we see that the B_1_ ion is now the base peak but *m/z* 430 and *m/z* 318 remained at less than 20% intensity.

MS^4^ of *m/z* 336 at CID 20 V in Figure 7D shows the formation of B_1_-H_2_O and a cross-ring cleavage at *m/z* 216. Other peaks appear at a higher CID energy of 35 V (Figure 6E).

The IT CID MS/MS of the [M + 2Na-H] ^+^ precursor ion was activated at lower energies in order to understand the formation of the product ions. For example, Supplemental Figure 3 shows the IT CID spectra of *m/z* 1043 of LSTa at increasing energies. At 22 V (Supplemental Figure 3A), *m/z* 905 and *m/z* 683 ions are starting to form. The B_3_-H_2_O ion (*m/z* 683) continues to rise in intensity at increasing energies (Supplemental Figure 3B–F). However, the ^2,4^A_5_-H_2_O (*m/z* 905) ion increased in intensity gradually near 40% at 25 V energy and then stayed near 20%, even though it started to appear early with *m/z* 683 at 22 V. The Y_4_-Na (*m/z* 730) ion increased in intensity with *m/z* 905 but surpassed the latter in the 25 V energy spectrum onwards.

We also probed at increasing HCD energies to see if the ions followed the same formation pattern, as shown in Supplemental Figure 4. At 20 V HCD energy (Supplemental Figure 4A), only *m/z* 905 and *m/z* 730 are observed at less than 5% intensity. More product ions appear at 25 V HCD energy (Supplemental Figure 4B). At 28 V HCD energy (Supplemental Figure 4C), *m/z* 730 is more abundant than *m/z* 683 and *m/z* 905. This is different from the CID spectrum, where *m/z* 683 is more abundant than *m/z* 730. Since the HCD activation fragments all the ions including the product ions, the B_1_ ion at *m/z* 336 should be viewed as an ion that comes from other ions that has a sialic acid, including B glycosidic ions and A cross-ring ions (*m/z* 498, 683, 905, 983). This might explain why *m/z* 730 (Y_4_-Na) ion is more abundant than *m/z* 683 (B_3_-H_2_O): some of the *m/z* 683 ions are further fragmented to produce *m/z* 336 (B_1_) ions in HCD.

**Figure 8:**
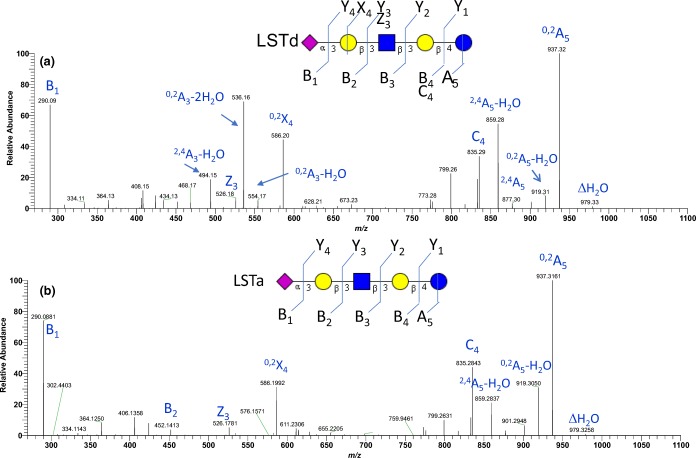
Consensus FT CID MS^2^ spectra of [M-H]- of two LST isomers (*m/z* 997.34): (**a**) LSTd (*N* = 146), (**b**) LSTa (*N* = 293)

The CID MS/MS analysis of LSTd and LSTa in the negative mode, shown in Figure 8, also suggests cross-ring cleavages. Of particular interest are *m/z* 536 and *m/z* 494 ions that are only present in LSTd (Figure 8A) and not LSTa (Figure 8B). The ions correspond to ^0,2^A_3_-2H_2_O and ^2,4^A_3_-H_2_O, respectively. These cross-ring fragmentations of negative ions are interesting; however, it is expected that the fragmentation mechanisms share not much similarities with the fragmentation mechanism of [M + 2Na-H] ^+^ ions, so will not extend the discussion on this topic. However, the presence of similar cross-ring fragmentation processes in the spectra of negative ions indicates that likely the effect of the sodium ions on the mass spectra of [M + 2Na-H] ^+^ is more related to the replacement of the acidic hydrogen by a sodium ion than to the promotion of the cross-ring fragmentation itself, whereas a second sodium ion coordinates several hydroxyl groups and adds rigidity to the structure.

A similar theoretical modeling strategy was designed for the study of the fragmentation mechanism of the [M + 2Na-H] ^+^ ions from LSTa and LSTc isomers. Supplemental Figure 5 shows the optimized structures for the two isomers. The absolute minima for isomer LSTa resulted in an extended structure similar to 3-SLN, and LSTc structure resembled the structure of 6-SLN. Although no further calculations were made for these isomers, a similar explanation probably holds for the observed differences of the ^2,4^A_5_ cross-ring fragmentation. It is worth mentioning that there is also some experimental data that supports the simultaneous coordination of cations to the glyceryl side chain and the carboxylate group [32, 33]. In general, the observed cross-ring fragmentation processes in [M + 2Na-H] ^+^ ions seem to follow certain general patterns: (i) a stabilizing effect of the gycosidic bond due to the replacement of the acidic hydrogen. Theoretical calculations show that one of the sodium ions is always near the carboxyl group at distances not larger than about 2.5 Å. (ii) The second sodium ion is also nearer to the non-reducing end in between the first and second rings, thus conferring certain rigidity to the structures. (iii) The fact that similar fragmentation processes are observed in the spectra of negative ions, [M-H]^−^, suggests a charge remote fragmentation mechanism. At this time, there is insufficient experimental and theoretical evidences to fully explain the role of the sodium atoms in the cross-ring fragmentation. (iv) Glycan branching and the presence of fucose in the reducing end inhibit the cross-ring fragmentation and the spectra can exhibit some unexpected peaks.

Lastly, we compared the QTOF CID and Orbitrap HCD MS/MS spectra of these isomers, as shown in Supplemental Figure 6 for LSTd and Supplemental Figure 7 for LSTb. The fragmentation patterns are very similar for most isomers. However, certain comparisons may require constraints, because the spectral differences among isomers are not very pronounced and noisy experimental spectra can match the wrong isomers. Table 2 shows an example of the library search results for the QTOF spectra of the isomer LSTb. It is observed that low collision energy spectra, with low conversion of the parent ion, match the wrong isomers (and with poor scores). A similar problem is observed at high collision energies. On the contrary, QTOF spectra with degree of the parent ion conversion between 5 and 90% match well with the HCD spectra in the library. This is the general finding in the NIST 17 library [34, 35] that spectra of the same precursor on different instruments are generally nearly identical at equivalent extents of decomposition (or effective collision energy). In practice, if library spectra are acquired at over a range of energies the include that of the query spectrum, the closely matching library spectra will appear at the top of the hit list and will have the closest matching fragmentation energy. We also note that when making identification by library searching one typically uses absolute scores (> 800 is usually a good match), differences between the top and the next best hit (100 is usually a good separation) [36, 37], although expertise concerning the fragmentation of the structural class under study is always an essential requirement for a confident identification.

**Table 2:** Comparison Between the QTOF CID and Orbitrap HCD MS/MS Spectra of [M + 2Na-H]^+^ Ions of LST Isomers

Experimental QTOF spectrum (isomer, spectrum type, NCE)	Orbitrap consensus spectrum Library match (isomer, spectrum type, NCE)	Score	Extent of fragmentation (%)
LSTb, CID, 40 V	LSTd, HCD, 63 V	688	< 1%
	LSTd, HCD, 65 V	687	
	LSTd, HCD, 61 V	668	
	LSTd, HCD, 60 V	661	
	LSTd, HCD, 58 V	649	
LSTb, CID, 50 V	LSTb, HCD, 68 V	767	≈ 5%
	LSTb, HCD, 70 V	767	
	LSTb, HCD, 73 V	763	
	LSTb, HCD, 66 V	761	
	LSTb, HCD, 65 V	761	
LSTb, CID, 60 V	LSTb, HCD, 73 V	868	≈ 50%
	LSTb, HCD, 70 V	864	
	LSTb, HCD, 68 V	857	
	LSTb, HCD, 66 V	845	
	LSTb, HCD, 83 V	842	
LSTb, CID, 70 V	LSTb, HCD, 84 V	909	≈ 90%
	LSTb, HCD, 70 V	889	
	LSTb, HCD, 93 V	872	
	LSTb, HCD, 73 V	868	
	LSTd, HCD, 83 V	864	
LSTb, CID, 90 V	LSTb, HCD, 104 V	938	> 99%
	LSTd, HCD, 103 V	932	
	LSTb, HCD, 114 V	915	
	LSTd, HCD, 112 V	915	
	LSTd, HCD, 93 V	913	

A downloadable NIST Glycan MS/MS library and MS Search software is available at https://chemdata.nist.gov/dokuwiki/doku.php?id=chemdata:glycan-library for enhanced search options. Finally, it is worth mentioning that the NIST library browser provides several tools that enhance the identification capabilities, such as hybrid and exact mass searches. For example, although the number of glycans in the library is relatively small similarity searches can match other related glycans.

## Conclusion

The fragmentation pattern of the [M + 2X-H]^+^ ion of sialyl isomers allowed us to differentiate spectra of underivatized sialyl isomers. The MS/MS spectra suggest that the main contribution to the spectral differentiation derive from an A cross-ring cleavage in the GlcNAc residue. We found that the differentiation is specific not only to the α2,3 and α2,6 NeuAc acid linkages, but to NeuAc-α2,3-Gal-β1,4-GlcNAc (or NeuAc-α2,3-Gal-β1,4-Glc) linkage, as we showed in the sialyllactose, sialylactosamine, and penta-sialyl isomers. To our knowledge, this is the first time that this observation is reported. Theoretical calculations show that the [M + 2Na-H]^+^ ions derived from glycans with α2,3 and α2,6 NeuAc acid linkages produce different conformational structures. The former linkage leads to rigid and extended structures, while the latter allows the molecule to bend on itself forming compact structures. These structural differences are consistent with the observed disparities between fragmentation patterns.

The NIST library of glycans contains MS/MS spectra from a broad range of collision energies and allows users to perform an exhaustive comparison and analysis of the differences between spectra, including relative small peak intensity differences. Consensus spectra searched against the library showed that the differentiation of isomers, at least in the case of [M + 2Na-H]^+^ ions of pentasaccharide sialyl isomers, was possible. It was also shown that for an effective comparison, the degree of parent ion conversion must be between 5 to 90%. Moreover, the CID QTOF and HCD Orbitrap MS/MS spectra of oligosaccharides showed good spectral match regardless of the type of mass spectrometer.

## Electronic Supplementary Material

ESM 1 (DOCX 1518 kb)

## Footnotes

1 ppm is conventionally used in mass spectrometry and corresponds to (μg/g)/charge.
