# Supplementary material for: Cross-Ring Fragmentation Patterns in the Tandem Mass Spectra of Underivatized Sialylated Oligosaccharides and Their Special Suitability for Spectrum Library Searching
Source: J Am Soc Mass Spectrom. 2018 Dec 18;30(3):426–38. doi: 10.1007/s13361-018-2106-8 (PMC6416239; doi:10.1007/s13361-018-2106-8)
Supplement: Supplementary file 1 — (DOCX 1518 kb) [file 13361_2018_2106_MOESM1_ESM.docx]

*Journal of the American Society for Mass Spectrometry*

**Supplemental Information**

Cross-ring fragmentation patterns in the tandem mass spectra of underivatized sialylated oligosaccharides and their special suitability for spectrum library searching

Maria Lorna A. De Leoz^1,*^, Yamil Simón-Manso^1^, Robert J. Woods^2^, Stephen E. Stein^1^

^1^National Institute of Standards & Technology, 100 Bureau Drive Stop 8362 Gaithersburg,

MD 20899, USA

^2^Complex Carbohydrate Research Center and Department of Biochemistry and Molecular Biology, University of Georgia, 315 Riverbend Road, Athens, GA 30602, USA

*To whom correspondence should be addressed: Maria Lorna A. De Leoz, Mass Spectrometry Data Center, National Institute of Standards and Technology, 100 Bureau Drive Gaithersburg, MD 20899-8362, Email: lornadeleoz@gmail.com, Tel: +1 (240) 630-2726, Fax: +1 (301) 975-2643.

**Supplemental Figures**

**Supplemental Figure 1.** Positive Fourier Transform collision-induced dissociation (FT CID) MS/MS spectra of [M+X]^+^ ion of 3-sialyllactose (SL) (left) and 6-SL, where X is A) lithium adducted to 3-SL, *m/z* 640.23; B) lithium adducted to 6-SL, *m/z* 640.23; C) sodium adducted to 3-SL, *m/z* 656.20; D) sodium adducted to 6-SL, *m/z* 656.20; E) potassium adducted to 3-SL, *m/z* 672.17; or F) potassium adducted to 6-SL, *m/z* 672.17.

**Supplemental Figure 2.** Density Functional Theory (DFT)-optimized structures of the ^2,4^A_3_ fragment ions from A) 3-sialyllactosamine (SLN) and B) 6-SLN.

**Supplemental Figure 3**. IT CID MS/MS Spectra of LSTa [M+2Na-H]^+^ precursor ion at *m/z* 1043 at increasing energies. A) 22V, B) 23V, C) 24V, D) 25V, E) 26V, F) 35V.

**Supplemental Figure 4.** FT HCD MS/MS spectra of LSTa at increasing collisional energies. A) 20V, B) 25V, C) 28V, D) 30V, E) 32V, F) 36V.

**Supplemental Figure 5.** Semi-empirical AM1 optimized structures of A) LSTa and B) LSTc.

**Supplemental Figure 6**. Orbitrap HCD and QToF CID MS/MS spectra across several energies of LSTd. All orbitrap spectra in the NIST 17 Tandem MS Library.

**Supplemental Figure 7**. Orbitrap HCD and QToF CID MS/MS spectra across several energies of LSTb. All orbitrap spectra in the NIST 17 Tandem MS Library.

**Supplemental Table**

**Supplemental Table 1.** Cartesian coordinates and bonding connectivity of doubly sodiated A) 3-sialyllactosamine and B) 6-sialyllactosamine. Format: mol file. Created by GaussView 5.0.9.

**Scheme S2.** The chemical structure and potential fragmentation mechanism of the ion [M+2Na-Fuc-H]+ at m/z 701 from SLeX (based on DFT/B3LYP calculations).


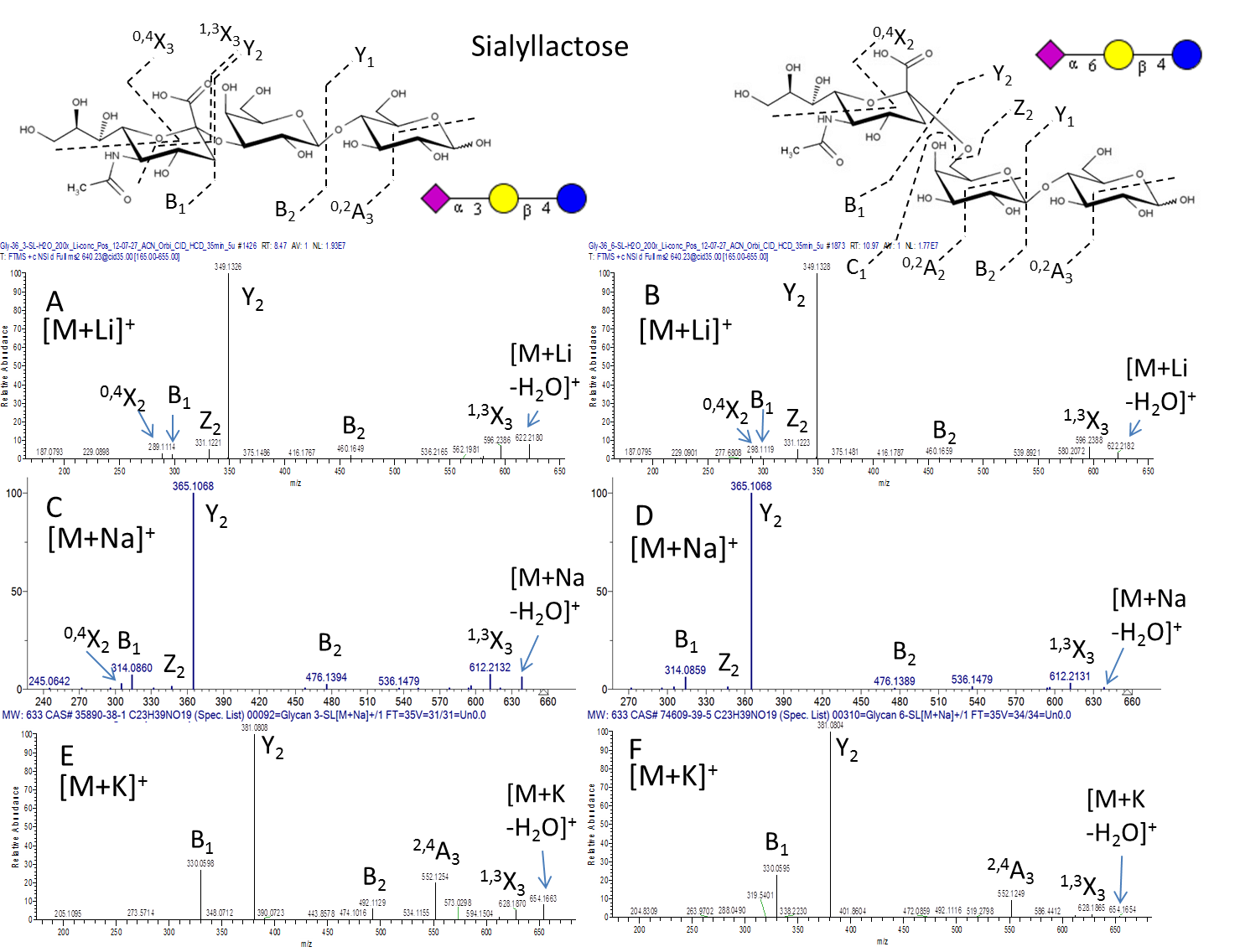
**Supplemental Figure 1.** Positive Fourier Transform collision-induced dissociation (FT CID) MS/MS spectra of [M+X]^+^ ion of 3-sialyllactose (SL) (left) and 6-SL (right), where X is A) lithium adducted to 3-SL, *m/z* 640.23; B) lithium adducted to 6-SL, *m/z* 640.23; C) sodium adducted to 3-SL, *m/z* 656.20; D) sodium adducted to 6-SL, *m/z* 656.20; E) potassium adducted to 3-SL, *m/z* 672.17; or F) potassium adducted to 6-SL, *m/z* 672.17.

**Supplemental Figure 2.** Density Functional Theory (DFT)-optimized structures of the ^2,4^A_3_ fragment ions from A) 3-sialyllactosamine (SLN) and B) 6-SLN.


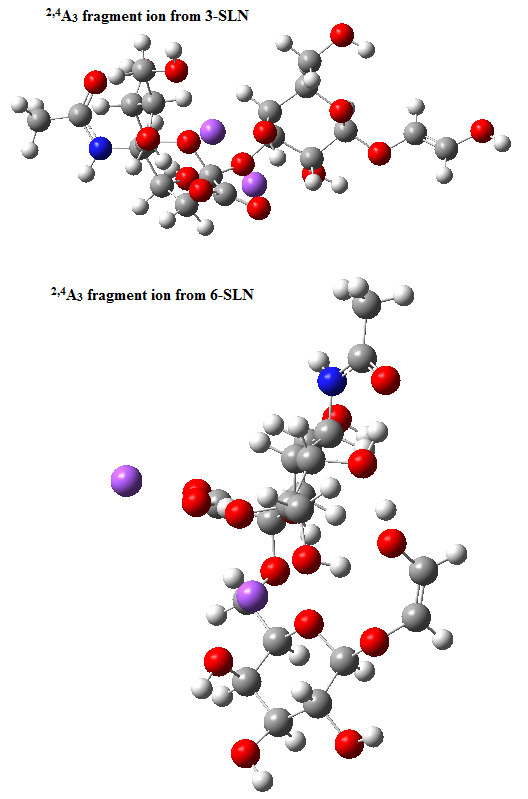


a

b

**Supplemental Figure 3**. IT CID MS/MS Spectra of LSTa [M+2Na-H]^+^ precursor ion at *m/z* 1043 at increasing energies. A) 22V, B) 23V, C) 24V, D) 25V, E) 26V, F) 35V.


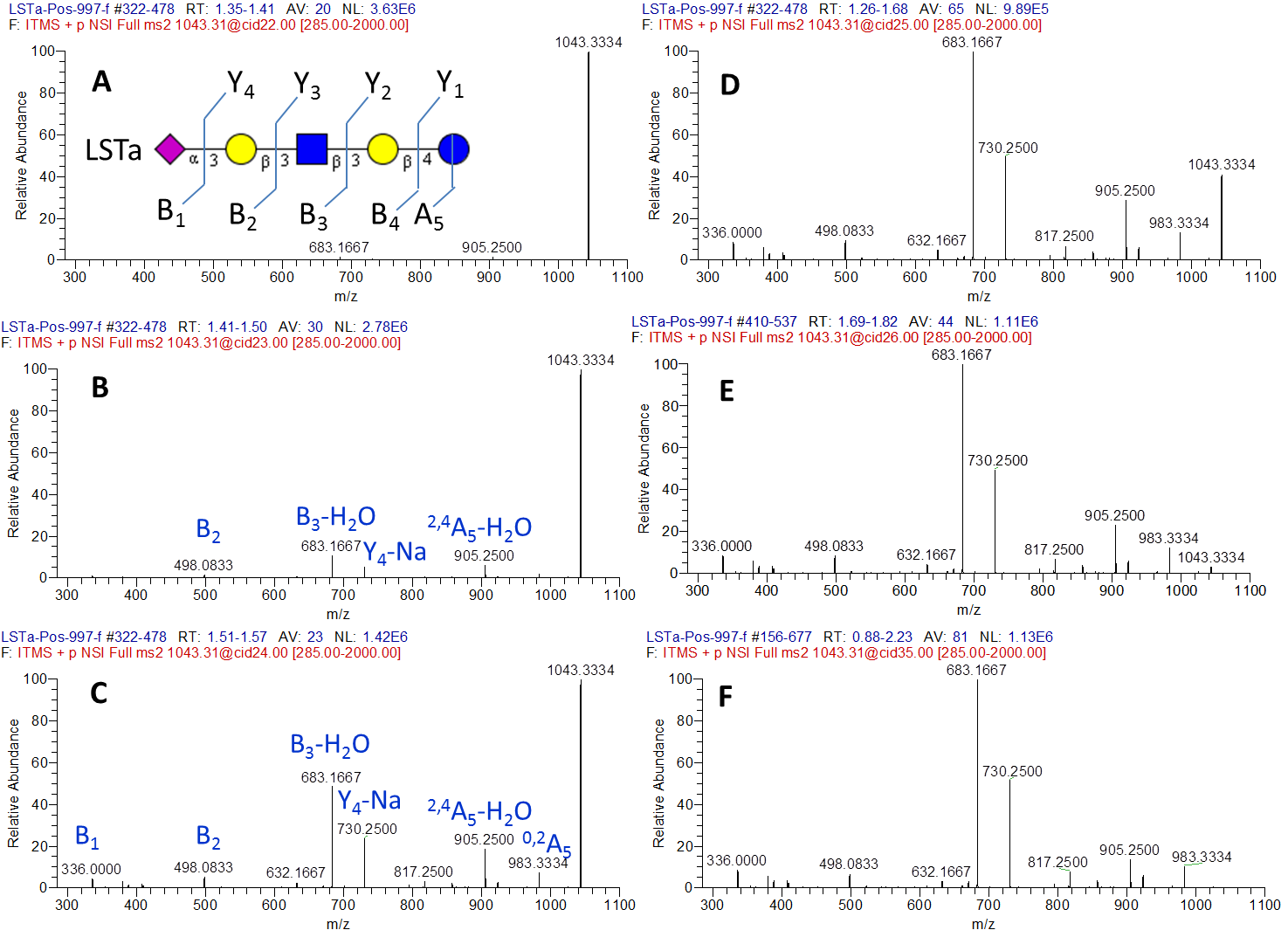


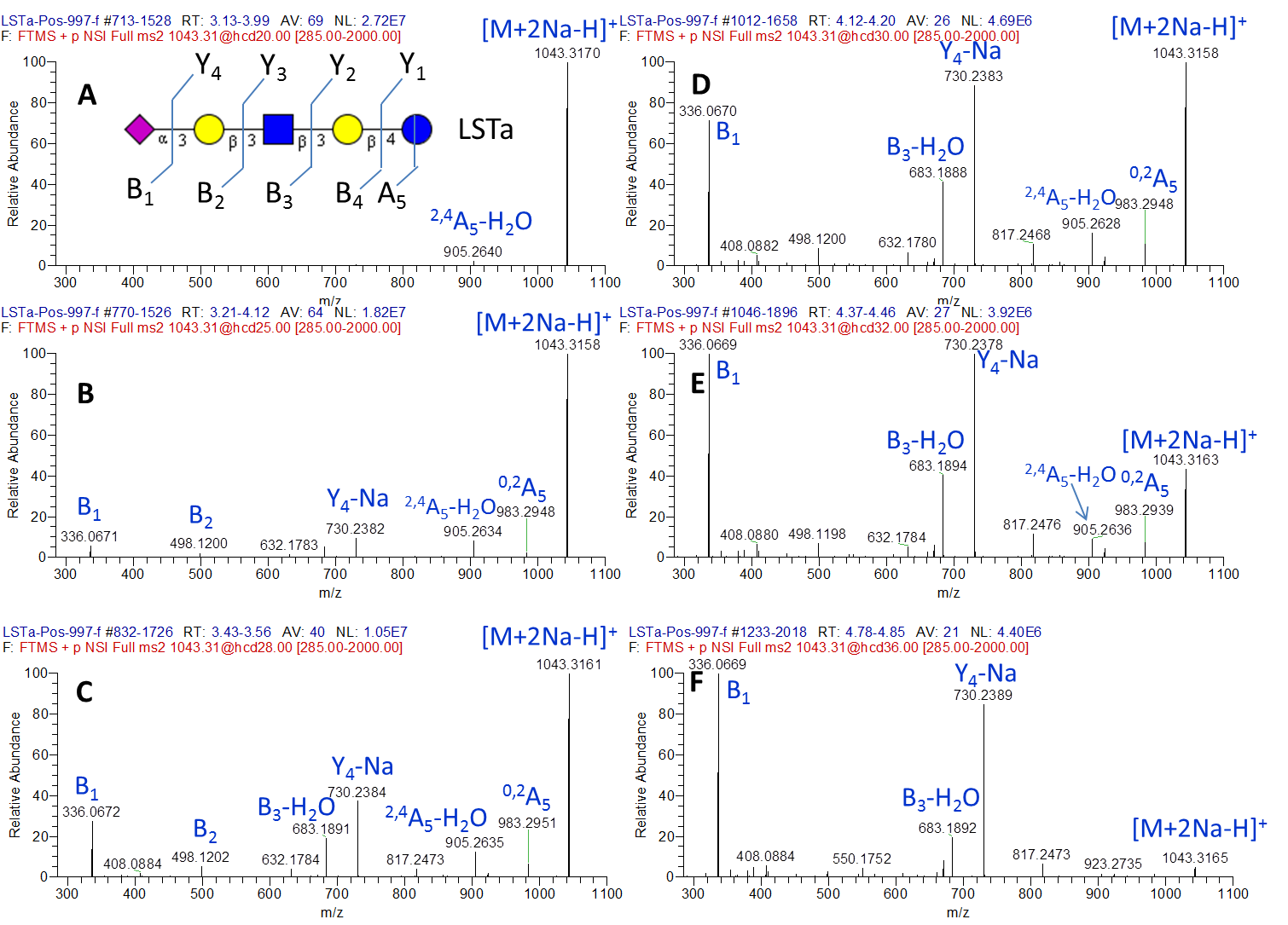
**Supplemental Figure 4.** FT HCD MS/MS spectra of LSTa at increasing collisional energies. A) 20V, B) 25V, C) 28V, D) 30V, E) 32V, F) 36V.

**Supplemental Figure 5.** Semi-empirical AM1 optimized structures of A) LSTa and B) LSTc.


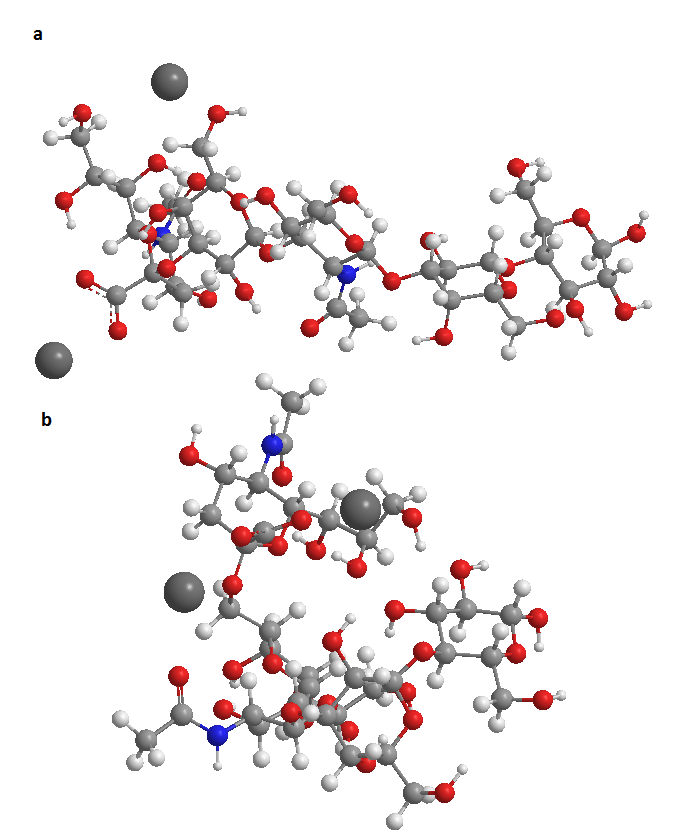


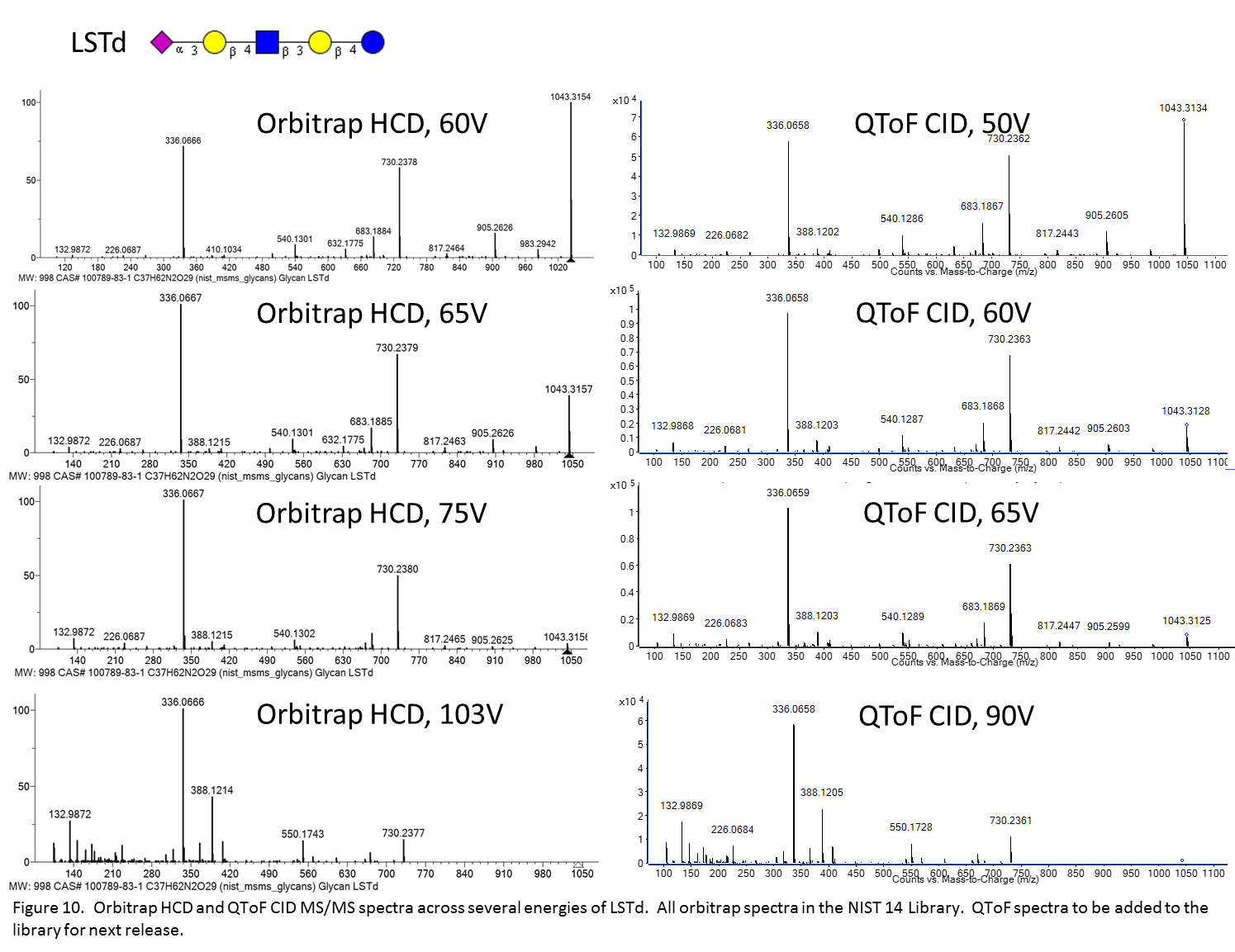
**Supplemental Figure 6**. Orbitrap HCD and QToF CID MS/MS spectra across several energies of LSTd. All orbitrap spectra in the NIST 17 Tandem MS Library.


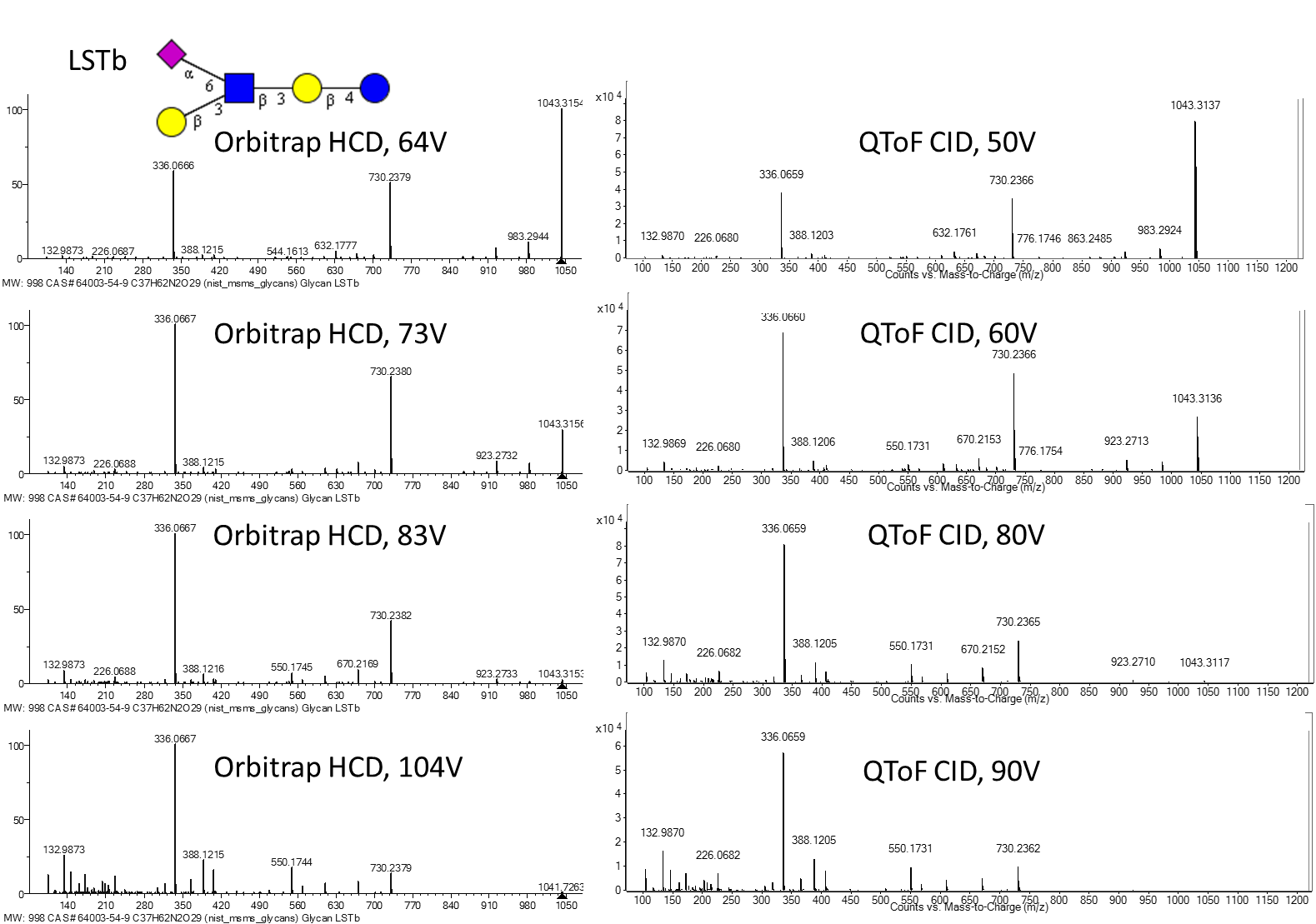
**Supplemental Figure 7**. Orbitrap HCD and QToF CID MS/MS spectra across several energies of LSTb. All orbitrap spectra in the NIST 17 Tandem MS Library.

**Supplemental Table 1.** Cartesian coordinates and bonding connectivity of doubly sodiated A) 3-sialyllactosamine and B) 6-sialyllactosamine. Format: mol file. Created by GaussView 5.0.9.

3-sln_2Na isomer

Created by GaussView 5.0.9

89 89 0 0 0 0 0 0 0 0 0 0

8.9530 -2.2500 0.4290 H 0 0 0 0 0 0 0 0 0 0 0 0

8.5880 -1.5180 -0.1100 O 0 0 0 0 0 0 0 0 0 0 0 0

7.2630 -1.1780 0.2900 C 0 0 0 0 0 0 0 0 0 0 0 0

7.2500 -0.7470 1.3090 H 0 0 0 0 0 0 0 0 0 0 0 0

6.4560 -2.3900 0.2930 O 0 0 0 0 0 0 0 0 0 0 0 0

5.1020 -2.1480 0.8020 C 0 0 0 0 0 0 0 0 0 0 0 0

5.1480 -1.8050 1.8500 H 0 0 0 0 0 0 0 0 0 0 0 0

4.4330 -3.5340 0.7120 C 0 0 0 0 0 0 0 0 0 0 0 0

5.0400 -4.2650 1.2580 H 0 0 0 0 0 0 0 0 0 0 0 0

4.3620 -3.8480 -0.3320 H 0 0 0 0 0 0 0 0 0 0 0 0

3.0530 -3.5590 1.2200 O 0 0 0 0 0 0 0 0 0 0 0 0

3.0270 -3.3680 2.1800 H 0 0 0 0 0 0 0 0 0 0 0 0

4.4090 -1.0630 -0.0440 C 0 0 0 0 0 0 0 0 0 0 0 0

4.1220 -1.4810 -1.0200 H 0 0 0 0 0 0 0 0 0 0 0 0

5.2510 0.2000 -0.3390 C 0 0 0 0 0 0 0 0 0 0 0 0

5.2760 0.8310 0.5650 H 0 0 0 0 0 0 0 0 0 0 0 0

4.5290 0.8460 -1.4130 O 0 0 0 0 0 0 0 0 0 0 0 0

5.0470 1.6670 -1.6740 H 0 0 0 0 0 0 0 0 0 0 0 0

6.7090 -0.1730 -0.7370 C 0 0 0 0 0 0 0 0 0 0 0 0

6.6800 -0.6780 -1.7140 H 0 0 0 0 0 0 0 0 0 0 0 0

7.6730 0.9520 -0.7760 N 0 0 0 0 0 0 0 0 0 0 0 0

8.6300 0.6600 -0.5940 H 0 0 0 0 0 0 0 0 0 0 0 0

7.4840 2.2140 -1.2740 C 0 0 0 0 0 0 0 0 0 0 0 0

6.3600 2.6460 -1.6720 O 0 0 0 0 0 0 0 0 0 0 0 0

8.7100 3.1070 -1.3260 C 0 0 0 0 0 0 0 0 0 0 0 0

8.9210 3.3600 -2.3710 H 0 0 0 0 0 0 0 0 0 0 0 0

8.4910 4.0440 -0.8050 H 0 0 0 0 0 0 0 0 0 0 0 0

9.6000 2.6490 -0.8830 H 0 0 0 0 0 0 0 0 0 0 0 0

3.2120 -0.7130 0.7250 O 0 0 0 0 0 0 0 0 0 0 0 0

2.0780 -0.2280 0.0110 C 0 0 0 0 0 0 0 0 0 0 0 0

2.2220 -0.3450 -1.0740 H 0 0 0 0 0 0 0 0 0 0 0 0

1.8870 1.1780 0.3260 O 0 0 0 0 0 0 0 0 0 0 0 0

0.7660 1.7420 -0.4490 C 0 0 0 0 0 0 0 0 0 0 0 0

0.8990 1.4900 -1.5140 H 0 0 0 0 0 0 0 0 0 0 0 0

0.8890 3.2590 -0.3020 C 0 0 0 0 0 0 0 0 0 0 0 0

0.9330 3.5190 0.7650 H 0 0 0 0 0 0 0 0 0 0 0 0

0.0310 3.7590 -0.7630 H 0 0 0 0 0 0 0 0 0 0 0 0

2.0540 3.7420 -1.0190 O 0 0 0 0 0 0 0 0 0 0 0 0

2.8510 3.2490 -0.7250 H 0 0 0 0 0 0 0 0 0 0 0 0

-0.5600 1.1350 0.0680 C 0 0 0 0 0 0 0 0 0 0 0 0

-1.3970 1.4850 -0.5420 H 0 0 0 0 0 0 0 0 0 0 0 0

-0.8410 1.6320 1.4170 O 0 0 0 0 0 0 0 0 0 0 0 0

-0.3840 1.0930 2.1030 H 0 0 0 0 0 0 0 0 0 0 0 0

-0.4390 -0.4150 -0.1030 C 0 0 0 0 0 0 0 0 0 0 0 0

-0.3740 -0.5400 -1.1960 H 0 0 0 0 0 0 0 0 0 0 0 0

0.8470 -1.0150 0.5010 C 0 0 0 0 0 0 0 0 0 0 0 0

0.7980 -0.9930 1.5950 H 0 0 0 0 0 0 0 0 0 0 0 0

0.9430 -2.4070 0.0360 O 0 0 0 0 0 0 0 0 0 0 0 0

1.7590 -2.8430 0.4350 H 0 0 0 0 0 0 0 0 0 0 0 0

-1.5480 -1.3200 0.2740 O 0 0 0 0 0 0 0 0 0 0 0 0

-2.7010 -0.9490 1.0640 C 0 0 0 0 0 0 0 0 0 0 0 0

-2.3070 -0.3320 2.4610 C 0 0 0 0 0 0 0 0 0 0 0 0

-3.1660 0.5190 2.9330 O 0 0 0 0 0 0 0 0 0 0 0 0

-1.1910 -0.6800 2.9610 O 0 0 0 0 0 0 0 0 0 0 0 0

-3.5510 -2.2240 1.2190 C 0 0 0 0 0 0 0 0 0 0 0 0

-4.2880 -2.0450 2.0110 H 0 0 0 0 0 0 0 0 0 0 0 0

-2.9270 -3.0530 1.5670 H 0 0 0 0 0 0 0 0 0 0 0 0

-4.3090 -2.6260 -0.0620 C 0 0 0 0 0 0 0 0 0 0 0 0

-5.0940 -3.3400 0.2300 H 0 0 0 0 0 0 0 0 0 0 0 0

-4.9710 -1.4180 -0.8170 C 0 0 0 0 0 0 0 0 0 0 0 0

-4.4070 -1.2200 -1.7360 H 0 0 0 0 0 0 0 0 0 0 0 0

-4.8510 -0.1450 0.0560 C 0 0 0 0 0 0 0 0 0 0 0 0

-5.3870 -0.2900 1.0060 H 0 0 0 0 0 0 0 0 0 0 0 0

-5.3120 1.2220 -0.5290 C 0 0 0 0 0 0 0 0 0 0 0 0

-6.4110 1.2730 -0.4790 H 0 0 0 0 0 0 0 0 0 0 0 0

-4.6520 2.2960 0.3670 C 0 0 0 0 0 0 0 0 0 0 0 0

-3.5820 2.1940 0.1460 H 0 0 0 0 0 0 0 0 0 0 0 0

-4.9820 3.7540 0.1120 C 0 0 0 0 0 0 0 0 0 0 0 0

-4.9500 3.9790 -0.9590 H 0 0 0 0 0 0 0 0 0 0 0 0

-5.9460 4.0330 0.5530 H 0 0 0 0 0 0 0 0 0 0 0 0

-3.8810 4.4710 0.8190 O 0 0 0 0 0 0 0 0 0 0 0 0

-3.9650 5.4390 0.7130 H 0 0 0 0 0 0 0 0 0 0 0 0

-4.9270 2.0550 1.7810 O 0 0 0 0 0 0 0 0 0 0 0 0

-4.4130 1.2720 2.1620 H 0 0 0 0 0 0 0 0 0 0 0 0

-4.8250 1.4630 -1.8680 O 0 0 0 0 0 0 0 0 0 0 0 0

-5.3290 0.9050 -2.5180 H 0 0 0 0 0 0 0 0 0 0 0 0

-3.4180 0.0410 0.3030 O 0 0 0 0 0 0 0 0 0 0 0 0

-6.3540 -1.7620 -1.1940 N 0 0 0 0 0 0 0 0 0 0 0 0

-6.8970 -2.3550 -0.5750 H 0 0 0 0 0 0 0 0 0 0 0 0

-6.9730 -1.2500 -2.3170 C 0 0 0 0 0 0 0 0 0 0 0 0

-8.3800 -1.7330 -2.6060 C 0 0 0 0 0 0 0 0 0 0 0 0

-8.7510 -2.4590 -1.8750 H 0 0 0 0 0 0 0 0 0 0 0 0

-8.4020 -2.1880 -3.6030 H 0 0 0 0 0 0 0 0 0 0 0 0

-9.0590 -0.8740 -2.6320 H 0 0 0 0 0 0 0 0 0 0 0 0

-6.3840 -0.4230 -3.0690 O 0 0 0 0 0 0 0 0 0 0 0 0

-3.3680 -3.3690 -0.9440 O 0 0 0 0 0 0 0 0 0 0 0 0

-3.8520 -3.7190 -1.7220 H 0 0 0 0 0 0 0 0 0 0 0 0

-2.7110 2.8810 2.3220 Na 0 0 0 0 0 0 0 0 0 0 0 0

-1.0550 -3.3560 -0.5290 Na 0 0 0 0 0 0 0 0 0 0 0 0

1 2 1 0 0 0 0

2 3 1 0 0 0 0

3 4 1 0 0 0 0

3 5 1 0 0 0 0

3 19 1 0 0 0 0

5 6 1 0 0 0 0

6 7 1 0 0 0 0

6 8 1 0 0 0 0

6 13 1 0 0 0 0

8 9 1 0 0 0 0

8 10 1 0 0 0 0

8 11 1 0 0 0 0

11 12 1 0 0 0 0

13 14 1 0 0 0 0

13 15 1 0 0 0 0

13 29 1 0 0 0 0

15 16 1 0 0 0 0

15 17 1 0 0 0 0

15 19 1 0 0 0 0

17 18 1 0 0 0 0

19 20 1 0 0 0 0

19 21 1 0 0 0 0

21 22 1 0 0 0 0

21 23 4 0 0 0 0

23 24 2 0 0 0 0

23 25 1 0 0 0 0

25 26 1 0 0 0 0

25 27 1 0 0 0 0

25 28 1 0 0 0 0

29 30 1 0 0 0 0

30 31 1 0 0 0 0

30 32 1 0 0 0 0

30 46 1 0 0 0 0

32 33 1 0 0 0 0

33 34 1 0 0 0 0

33 35 1 0 0 0 0

33 40 1 0 0 0 0

35 36 1 0 0 0 0

35 37 1 0 0 0 0

35 38 1 0 0 0 0

38 39 1 0 0 0 0

40 41 1 0 0 0 0

40 42 1 0 0 0 0

40 44 1 0 0 0 0

42 43 1 0 0 0 0

44 45 1 0 0 0 0

44 46 1 0 0 0 0

44 50 1 0 0 0 0

46 47 1 0 0 0 0

46 48 1 0 0 0 0

48 49 1 0 0 0 0

50 51 1 0 0 0 0

51 52 1 0 0 0 0

51 55 1 0 0 0 0

51 77 1 0 0 0 0

52 53 4 0 0 0 0

52 54 2 0 0 0 0

55 56 1 0 0 0 0

55 57 1 0 0 0 0

55 58 1 0 0 0 0

58 59 1 0 0 0 0

58 60 1 0 0 0 0

58 86 1 0 0 0 0

60 61 1 0 0 0 0

60 62 1 0 0 0 0

60 78 1 0 0 0 0

62 63 1 0 0 0 0

62 64 1 0 0 0 0

62 77 1 0 0 0 0

64 65 1 0 0 0 0

64 66 1 0 0 0 0

64 75 1 0 0 0 0

66 67 1 0 0 0 0

66 68 1 0 0 0 0

66 73 1 0 0 0 0

68 69 1 0 0 0 0

68 70 1 0 0 0 0

68 71 1 0 0 0 0

71 72 1 0 0 0 0

73 74 1 0 0 0 0

75 76 1 0 0 0 0

78 79 1 0 0 0 0

78 80 4 0 0 0 0

80 81 1 0 0 0 0

80 85 2 0 0 0 0

81 82 1 0 0 0 0

81 83 1 0 0 0 0

81 84 1 0 0 0 0

86 87 1 0 0 0 0

B)

6-sln_2Na isomer

Created by GaussView 5.0.9

89 89 0 0 0 0 0 0 0 0 0 0

-0.6560 -6.1500 -1.9090 H 0 0 0 0 0 0 0 0 0 0 0 0

-0.1580 -5.4760 -1.4000 O 0 0 0 0 0 0 0 0 0 0 0 0

-1.0230 -4.6910 -0.6000 C 0 0 0 0 0 0 0 0 0 0 0 0

-1.5610 -5.3020 0.1520 H 0 0 0 0 0 0 0 0 0 0 0 0

-2.0270 -4.0810 -1.4800 O 0 0 0 0 0 0 0 0 0 0 0 0

-2.9670 -3.1790 -0.7920 C 0 0 0 0 0 0 0 0 0 0 0 0

-3.5180 -3.7400 -0.0180 H 0 0 0 0 0 0 0 0 0 0 0 0

-3.9490 -2.7430 -1.8850 C 0 0 0 0 0 0 0 0 0 0 0 0

-3.4020 -2.2000 -2.6730 H 0 0 0 0 0 0 0 0 0 0 0 0

-4.7320 -2.1020 -1.4720 H 0 0 0 0 0 0 0 0 0 0 0 0

-4.6470 -3.8910 -2.4310 O 0 0 0 0 0 0 0 0 0 0 0 0

-3.9890 -4.5300 -2.7810 H 0 0 0 0 0 0 0 0 0 0 0 0

-2.1520 -2.0460 -0.1190 C 0 0 0 0 0 0 0 0 0 0 0 0

-1.5800 -1.4830 -0.8680 H 0 0 0 0 0 0 0 0 0 0 0 0

-1.1650 -2.6740 0.8970 C 0 0 0 0 0 0 0 0 0 0 0 0

-1.7440 -3.3030 1.5930 H 0 0 0 0 0 0 0 0 0 0 0 0

-0.4990 -1.6830 1.6930 O 0 0 0 0 0 0 0 0 0 0 0 0

0.4930 -1.5640 1.5480 H 0 0 0 0 0 0 0 0 0 0 0 0

-0.1930 -3.5690 0.0660 C 0 0 0 0 0 0 0 0 0 0 0 0

0.2420 -2.9760 -0.7470 H 0 0 0 0 0 0 0 0 0 0 0 0

0.9400 -4.0870 0.8200 N 0 0 0 0 0 0 0 0 0 0 0 0

0.7770 -4.7370 1.5800 H 0 0 0 0 0 0 0 0 0 0 0 0

2.2430 -3.8770 0.3930 C 0 0 0 0 0 0 0 0 0 0 0 0

2.5100 -3.0970 -0.5610 O 0 0 0 0 0 0 0 0 0 0 0 0

3.3210 -4.6180 1.1600 C 0 0 0 0 0 0 0 0 0 0 0 0

2.9300 -5.2060 1.9970 H 0 0 0 0 0 0 0 0 0 0 0 0

3.8450 -5.2920 0.4710 H 0 0 0 0 0 0 0 0 0 0 0 0

4.0490 -3.8920 1.5360 H 0 0 0 0 0 0 0 0 0 0 0 0

-3.0680 -1.1140 0.5730 O 0 0 0 0 0 0 0 0 0 0 0 0

-3.3430 0.0740 -0.1490 C 0 0 0 0 0 0 0 0 0 0 0 0

-3.5020 -0.1330 -1.2200 H 0 0 0 0 0 0 0 0 0 0 0 0

-2.1950 1.0000 -0.0430 O 0 0 0 0 0 0 0 0 0 0 0 0

-2.3930 2.1480 -0.9520 C 0 0 0 0 0 0 0 0 0 0 0 0

-2.6600 1.7760 -1.9530 H 0 0 0 0 0 0 0 0 0 0 0 0

-3.5710 2.9700 -0.3990 C 0 0 0 0 0 0 0 0 0 0 0 0

-3.7920 3.8230 -1.0540 H 0 0 0 0 0 0 0 0 0 0 0 0

-3.1990 3.4780 0.9360 O 0 0 0 0 0 0 0 0 0 0 0 0

-4.0010 3.9110 1.3080 H 0 0 0 0 0 0 0 0 0 0 0 0

-4.8360 2.1020 -0.2570 C 0 0 0 0 0 0 0 0 0 0 0 0

-5.2400 1.8720 -1.2540 H 0 0 0 0 0 0 0 0 0 0 0 0

-5.7700 2.9240 0.4970 O 0 0 0 0 0 0 0 0 0 0 0 0

-6.6280 2.4630 0.6060 H 0 0 0 0 0 0 0 0 0 0 0 0

-4.5720 0.7640 0.4600 C 0 0 0 0 0 0 0 0 0 0 0 0

-4.3780 0.9320 1.5300 H 0 0 0 0 0 0 0 0 0 0 0 0

-5.7860 -0.0110 0.2730 O 0 0 0 0 0 0 0 0 0 0 0 0

-5.7690 -0.8260 0.8180 H 0 0 0 0 0 0 0 0 0 0 0 0

-1.0920 2.9360 -1.0750 C 0 0 0 0 0 0 0 0 0 0 0 0

-0.6320 3.0970 -0.1000 H 0 0 0 0 0 0 0 0 0 0 0 0

-1.3110 3.9140 -1.5270 H 0 0 0 0 0 0 0 0 0 0 0 0

-0.1750 2.2150 -1.9740 O 0 0 0 0 0 0 0 0 0 0 0 0

1.2070 2.1280 -1.5630 C 0 0 0 0 0 0 0 0 0 0 0 0

1.7670 3.5590 -1.2560 C 0 0 0 0 0 0 0 0 0 0 0 0

1.5930 4.0310 -0.0410 O 0 0 0 0 0 0 0 0 0 0 0 0

2.3550 4.2310 -2.1760 O 0 0 0 0 0 0 0 0 0 0 0 0

1.9870 1.3580 -2.6280 C 0 0 0 0 0 0 0 0 0 0 0 0

2.1180 1.9650 -3.5270 H 0 0 0 0 0 0 0 0 0 0 0 0

1.3970 0.4670 -2.8780 H 0 0 0 0 0 0 0 0 0 0 0 0

3.3750 0.9550 -2.0860 C 0 0 0 0 0 0 0 0 0 0 0 0

3.9830 1.8640 -1.9840 H 0 0 0 0 0 0 0 0 0 0 0 0

3.3420 0.2330 -0.7000 C 0 0 0 0 0 0 0 0 0 0 0 0

2.8510 -0.7470 -0.7850 H 0 0 0 0 0 0 0 0 0 0 0 0

2.5230 1.1180 0.2840 C 0 0 0 0 0 0 0 0 0 0 0 0

3.0440 2.0770 0.4260 H 0 0 0 0 0 0 0 0 0 0 0 0

2.1810 0.5400 1.6880 C 0 0 0 0 0 0 0 0 0 0 0 0

2.9880 0.7970 2.3890 H 0 0 0 0 0 0 0 0 0 0 0 0

0.8080 1.0740 2.1510 C 0 0 0 0 0 0 0 0 0 0 0 0

0.1130 0.6650 1.4120 H 0 0 0 0 0 0 0 0 0 0 0 0

0.3520 0.5790 3.5300 C 0 0 0 0 0 0 0 0 0 0 0 0

0.8670 -0.3410 3.8180 H 0 0 0 0 0 0 0 0 0 0 0 0

0.5040 1.3480 4.2920 H 0 0 0 0 0 0 0 0 0 0 0 0

-1.1060 0.3300 3.4570 O 0 0 0 0 0 0 0 0 0 0 0 0

-1.2110 -0.5190 2.9440 H 0 0 0 0 0 0 0 0 0 0 0 0

0.6670 2.5410 2.1560 O 0 0 0 0 0 0 0 0 0 0 0 0

1.0450 2.9910 1.3460 H 0 0 0 0 0 0 0 0 0 0 0 0

2.0070 -0.9040 1.6550 O 0 0 0 0 0 0 0 0 0 0 0 0

2.9280 -1.3170 1.5770 H 0 0 0 0 0 0 0 0 0 0 0 0

1.2120 1.3540 -0.3400 O 0 0 0 0 0 0 0 0 0 0 0 0

4.7630 0.0410 -0.3210 N 0 0 0 0 0 0 0 0 0 0 0 0

5.4300 0.3570 -1.0210 H 0 0 0 0 0 0 0 0 0 0 0 0

5.2440 -0.8280 0.6160 C 0 0 0 0 0 0 0 0 0 0 0 0

6.7460 -1.0410 0.6430 C 0 0 0 0 0 0 0 0 0 0 0 0

7.2920 -0.3380 0.0060 H 0 0 0 0 0 0 0 0 0 0 0 0

6.9640 -2.0620 0.3080 H 0 0 0 0 0 0 0 0 0 0 0 0

7.1050 -0.9510 1.6730 H 0 0 0 0 0 0 0 0 0 0 0 0

4.4980 -1.4450 1.4390 O 0 0 0 0 0 0 0 0 0 0 0 0

4.1300 0.1650 -3.0510 O 0 0 0 0 0 0 0 0 0 0 0 0

3.6690 -0.6780 -3.2540 H 0 0 0 0 0 0 0 0 0 0 0 0

2.6150 6.0090 -0.7310 Na 0 0 0 0 0 0 0 0 0 0 0 0

-1.7080 2.1950 2.2000 Na 0 0 0 0 0 0 0 0 0 0 0 0

1 2 1 0 0 0 0

2 3 1 0 0 0 0

3 4 1 0 0 0 0

3 5 1 0 0 0 0

3 19 1 0 0 0 0

5 6 1 0 0 0 0

6 7 1 0 0 0 0

6 8 1 0 0 0 0

6 13 1 0 0 0 0

8 9 1 0 0 0 0

8 10 1 0 0 0 0

8 11 1 0 0 0 0

11 12 1 0 0 0 0

13 14 1 0 0 0 0

13 15 1 0 0 0 0

13 29 1 0 0 0 0

15 16 1 0 0 0 0

15 17 1 0 0 0 0

15 19 1 0 0 0 0

17 18 1 0 0 0 0

19 20 1 0 0 0 0

19 21 1 0 0 0 0

21 22 1 0 0 0 0

21 23 1 0 0 0 0

23 24 2 0 0 0 0

23 25 1 0 0 0 0

25 26 1 0 0 0 0

25 27 1 0 0 0 0

25 28 1 0 0 0 0

29 30 1 0 0 0 0

30 31 1 0 0 0 0

30 32 1 0 0 0 0

30 43 1 0 0 0 0

32 33 1 0 0 0 0

33 34 1 0 0 0 0

33 35 1 0 0 0 0

33 47 1 0 0 0 0

35 36 1 0 0 0 0

35 37 1 0 0 0 0

35 39 1 0 0 0 0

37 38 1 0 0 0 0

39 40 1 0 0 0 0

39 41 1 0 0 0 0

39 43 1 0 0 0 0

41 42 1 0 0 0 0

43 44 1 0 0 0 0

43 45 1 0 0 0 0

45 46 1 0 0 0 0

47 48 1 0 0 0 0

47 49 1 0 0 0 0

47 50 1 0 0 0 0

50 51 1 0 0 0 0

51 52 1 0 0 0 0

51 55 1 0 0 0 0

51 77 1 0 0 0 0

52 53 4 0 0 0 0

52 54 2 0 0 0 0

55 56 1 0 0 0 0

55 57 1 0 0 0 0

55 58 1 0 0 0 0

58 59 1 0 0 0 0

58 60 1 0 0 0 0

58 86 1 0 0 0 0

60 61 1 0 0 0 0

60 62 1 0 0 0 0

60 78 1 0 0 0 0

62 63 1 0 0 0 0

62 64 1 0 0 0 0

62 77 1 0 0 0 0

64 65 1 0 0 0 0

64 66 1 0 0 0 0

64 75 1 0 0 0 0

66 67 1 0 0 0 0

66 68 1 0 0 0 0

66 73 1 0 0 0 0

68 69 1 0 0 0 0

68 70 1 0 0 0 0

68 71 1 0 0 0 0

71 72 1 0 0 0 0

73 74 1 0 0 0 0

75 76 1 0 0 0 0

78 79 1 0 0 0 0

78 80 4 0 0 0 0

80 81 1 0 0 0 0

80 85 2 0 0 0 0

81 82 1 0 0 0 0

81 83 1 0 0 0 0

81 84 1 0 0 0 0

86 87 1 0 0 0 0

**Frequency calculations using Gaussian 09 are available upon request.**

**
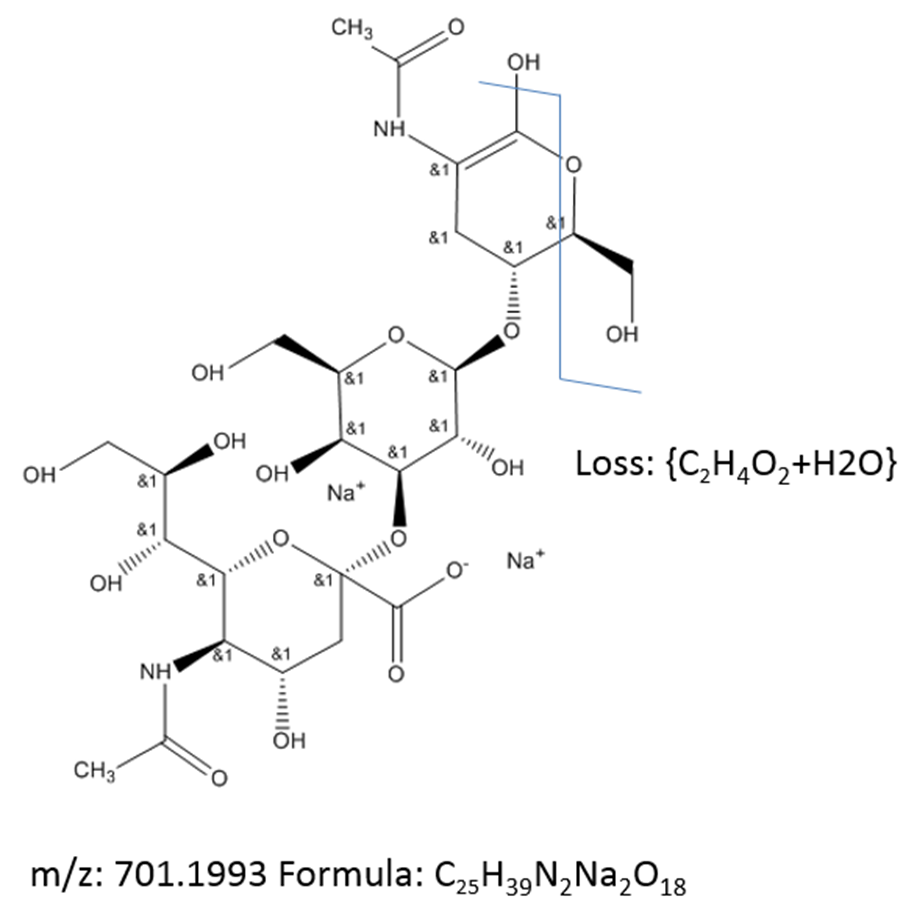
**
